# Striatal Neurons Are Recruited Dynamically into Collective Representations of Self-Initiated and Learned Actions in Freely Moving Mice

**DOI:** 10.1523/ENEURO.0315-23.2023

**Published:** 2024-01-10

**Authors:** Lior Tiroshi, Yara Atamna, Naomi Gilin, Noa Berkowitz, Joshua A. Goldberg

**Affiliations:** Department of Medical Neurobiology, Institute of Medical Research Israel – Canada, The Faculty of Medicine, The Hebrew University of Jerusalem, 9112102, Jerusalem, Israel

**Keywords:** basal ganglia, calcium imaging, correlations, parvalbumin-positive fast-spiking interneurons, population coding, spiny projection neurons

## Abstract

Striatal spiny projection neurons are hyperpolarized-at-rest (HaR) and driven to action potential threshold by a small number of powerful inputs—an input–output configuration that is detrimental to response reliability. Because the striatum is important for habitual behaviors and goal-directed learning, we conducted a microendoscopic imaging in freely moving mice that express a genetically encoded Ca^2+^ indicator sparsely in striatal HaR neurons to evaluate their response reliability during self-initiated movements and operant conditioning. The sparse expression was critical for longitudinal studies of response reliability, and for studying correlations among HaR neurons while minimizing spurious correlations arising from contamination by the background signal. We found that HaR neurons are recruited dynamically into action representation, with distinct neuronal subsets being engaged in a moment-by-moment fashion. While individual neurons respond with little reliability, the population response remained stable across days. Moreover, we found evidence for the temporal coupling between neuronal subsets during conditioned (but not innate) behaviors.

## Significance Statement

We explored the reliability of neuronal responses in the striatum by conducting microendoscopic imaging in transgenic mice expressing the Ca^2+^ indicator GCaMP6f sparsely in striatal neurons. The sparse expression allowed us to closely monitor individual neurons within sessions spanning days and weeks and estimate the reliability of their responses. We conclude that striatal neurons are recruited dynamically in response to both spontaneous and learned actions, exhibiting low reliability. Additionally, the sparseness enabled us to credibly measure trial-by-trial covariation among imaged neurons, yielding very little evidence for coordinated responses and arguing against the formation of spatially compact SPN clusters. Thus, the sparse expression of the indicator uncovered previously unobserved aspects of striatal collective responses.

## Introduction

A critical role of the central nervous system (CNS) is to maintain a robust and reliable representation of the external world and internal state of the organism. The degree of esponse reliability within a brain region determines the resources required to represent the external world and encode actions (Bullock, 1970; Shadlen and Newsome, 1998; Nolte et al., 2019), shaping the neural code (Mainen and Seinowski, 1995; Gallant and Vinje, 2001).

Considering the relationship between the size of the neuron’s individual synaptic inputs (Gutkin et al., 2003; Hunter and Milton, 2003) and the depolarization required to reach action potential (AP) threshold (Naundorf et al., 2006; Schreiber et al., 2009; Tchumatchenko et al., 2011; Puelma Touzel and Wolf, 2015) reveals diverse scenarios even within the same brain region. In one scenario, the neuron’s membrane potential is close to AP threshold (or the neuron is a pacemaker approaching AP threshold autonomously), and individual inputs are weak (Naundorf et al., 2006; Tchumatchenko et al., 2011; Puelma Touzel and Wolf, 2015). Here, an external event or action is encoded by numerous (even thousands of) afferent inputs, effectively averaging out noise and yielding a high-fidelity representation to which the neuron is expected to respond reliably. In another scenario, a neuron has a very hyperpolarized resting membrane potential but receives larger afferent inputs. With fewer inputs needed to reach AP threshold, there is less averaging out of input variability, potentially leading the responding neuron to represent the event or action with reduced reliability.

In the striatum—a major basal ganglia input nucleus integrating information from a wide range of brain regions (Yeterian and Van Hoesen, 1978; Flaherty and Graybiel, 1993; McFarland and Haber, 2000; Smith et al., 2004; Doig et al., 2010; Hunnicutt et al., 2016)—both scenarios exist. The primary inputs to the striatum are excitatory projections from the cortex and thalamus, with inputs from various cortical and thalamic sub-regions converging onto the same neurons (Inase et al., 1996; Takada et al., 1998a,b; McFarland and Haber, 2000). Cholinergic interneurons (CINs) and somatostatin-positive low-threshold spiking interneurons (LTSIs), two spontaneously active striatal populations (Bennett et al., 2000; Tepper and Koós, 2016)—whose voltage trajectory is driven autonomously to AP threshold—receive weak cortical and thalamic inputs (Johansson and Silberberg, 2020). In contrast, spiny projection neurons (SPNs), the majority of striatal neurons (Graveland and Difiglia, 1985; Gerfen, 1988; Tepper et al., 2008), and parvalbumin-positive (PV) interneurons that shape striatal output through the powerful inhibition of SPNs (Assous and Tepper, 2018) are all hyperpolarized-at-rest (HaR) and receive stronger inputs from these structures (Johansson and Silberberg, 2020). Therefore, the striatum provides a good testing ground for the relationship between input integration by individual neurons and their response reliability: autonomous pacemakers (CINs and LTSIs) should be highly reliable in their representation of stimuli and actions, while HaR neurons (SPNs and PV interneurons, the focus of the current study) should be less reliable. Indeed, striatal tonically active neurons (TANs), mostly comprised of CINs (Aosaki et al., 1995), exhibit a stereotypic burst response to salient inputs. Not only does the vast majority (50–90%) of TANs respond to the salient input, but individual trials reliably replicate the mean population response (Kimura et al., 1984; Raz et al., 1996; Apicella et al., 1997).

Based on the electrophysiological and functional connectivity properties of HaR neurons, we hypothesized that their overall reliability is low. To test this hypothesis, we performed microendoscopic Ca^2+^ imaging in freely moving mice, using a transgenic mouse strain expressing the Ca^2+^ indicator GCaMP6f (Chen et al., 2013) in a sparse population comprised almost exclusively of SPNs and PV interneurons—two HaR striatal subtypes that receive strong cortical and thalamic innervation (Johansson and Silberberg, 2020). We evaluated striatal HaR reliability during spontaneous movement and operant conditioning, expecting higher reliability for learned responses, whose behavioral value is presumably higher. The sparse expression of the Ca^2+^ indicator in our transgenic mice allowed us to compare individual neurons’ responses across sessions spanning days and weeks, revealing that while the population response remains stable across sessions, individual neurons respond with little reliability. Hypothesizing that the low reliability of individual neurons is compensated for by the formation of co-active clusters around a given action or stimulus, we leveraged our ability to simultaneously image dozens of isolated striatal neurons to explore the correlations between their signals. Focusing on a sparse population allowed for a clear separation between sources, revealing low, distance-independent pairwise correlations, in contrast to previous studies in dense SPN populations (Barbera et al., 2016; Parker et al., 2018; Shin et al., 2020).

## Materials and Methods

### Animals

All animal procedures were performed in accordance with the Hebrew University’s Institutional Animal Care and Use Committee’s regulations. Two- to 7-month-old mice were used in the experiments. Due to their larger size, male mice better tolerated the surgical procedures and prolonged food restriction and were therefore exclusively used. Animals were housed in a reversed light/dark facility.

**Figure 1.**
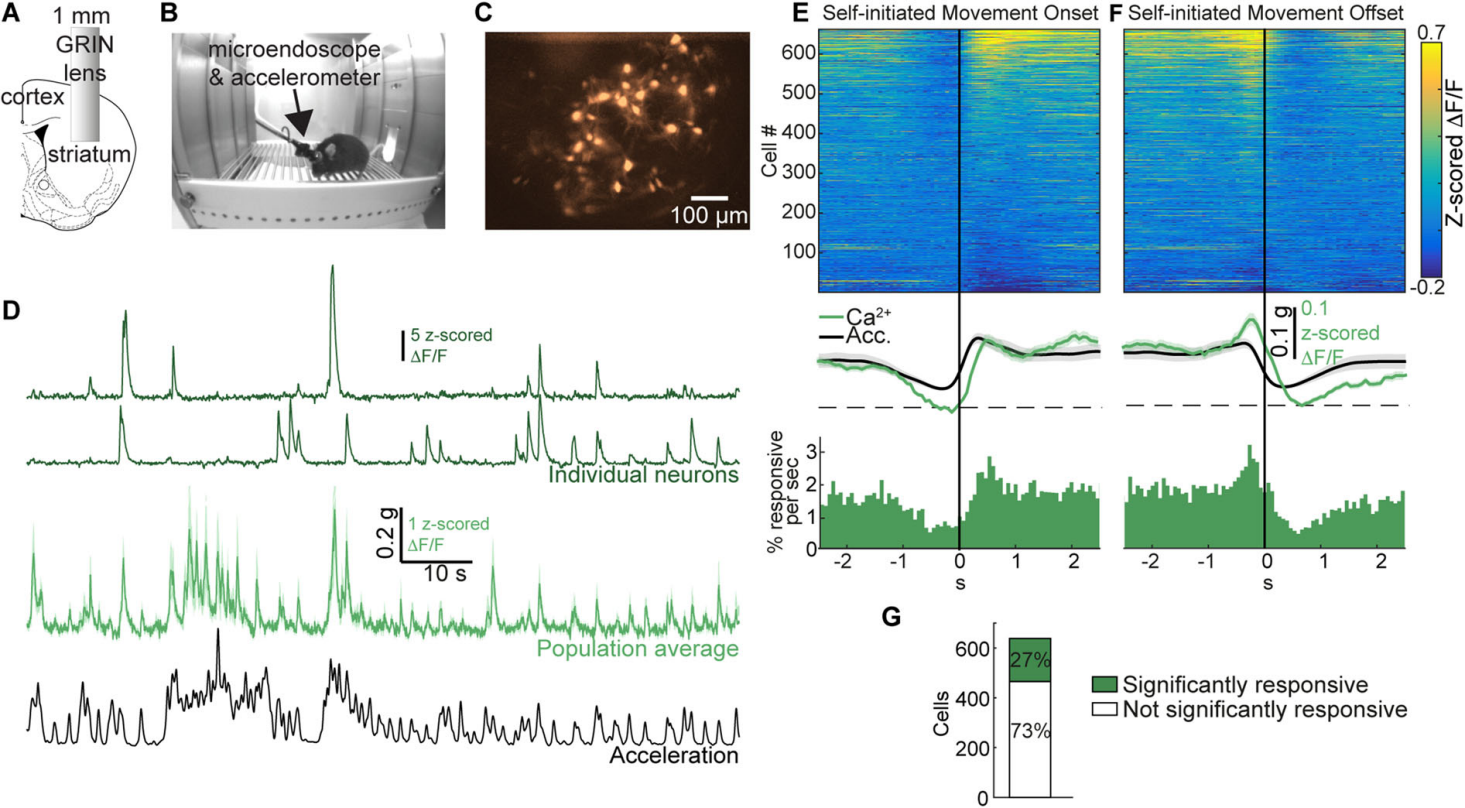
The collective neuronal activity is strongly modulated by self-initiated movement in freely moving mice. ***A***, A 1-mm-diameter GRIN lens is implanted into the dorsolateral striatum. ***B***, Implanted mouse with a microendoscope and an accelerometer mounted on its head moves freely in a behavior chamber. ***C***, Image via lens in freely moving mouse reveals signals from dozens of striatal neurons (see Fig. 1-1 for distribution of striatal subtypes). ***D***, Ca^2+^ signals of two individual neurons (top, green), the average Ca^2+^ signal across all imaged neurons (56 neurons, middle, green) and the simultaneously recorded total body acceleration of a representative mouse (bottom, black). The two individual neuron exhibited were chosen because they were both shown to respond significantly to changes in the acceleration: with the top one reducing its Ca^2+^ signal and bottom one increasing its Ca^2+^ signal. ***E***, Color-coded matrix showing activity around movement onset (Fig. 1-2), averaged across all movement onset events (top). Average Ca^2+^ activity across the population of imaged neurons and average total body acceleration across movement onset events are represented by green and black traces, respectively (middle). PSTH of Ca^2+^ events centered around the movement onset (bottom). Bin width: 67 ms. See Fig. 1-3 for description of positively and negatively modulated neurons. ***F***, Same as E but around movement offset. ***G***, Rates of detected neurons that did (green) or did not (white) significantly modulate their Ca^2+^ signals following movement onset (Fig. 1-2). Shaded areas represent S.E.M. g in scale bar is the gravitational constant 9.81 m/s^2^.

Transgenic mice that express Cre-recombinase sparsely in the striatum (stock number 006410; Jackson Laboratories) were cross-bred with Cre-dependent, Tet-controllable, GcaMP6f (Ai148, TIT2L-GC6f-ICL-tTA2) mice (stock 030328; Jackson Laboratories) to generate a sparse GCaMP mouse. The population of GcaMP6f-expressing neurons was dominated by SPNs (∼60%), with a moderate GABAergic interneuron population (∼30%) consisting mostly of PV interneurons, and a smaller proportion of CINs (Fig. 1-1).

### Gradient refractive index lens implantation

Eight- to 14-week-old mice were deeply anesthetized with isoflurane in a nonrebreathing system and placed in the stereotaxic frame. The temperature was maintained at 35°C with a heating pad, artificial tears were applied to prevent corneal drying, and animals were hydrated with a bolus of injectable saline (5 ml/kg) mixed with an analgesic (5 mg/kg carprofen). A bolus of 33 mg/kg of a ketamine–xylazine mixture was injected initially to stabilize the preparation for induction of anesthesia. A hole slightly wider than the 1-mm-diameter (4-mm-long) gradient refractive index (GRIN) lens was drilled into the skull in aseptic conditions. The hole was centered around the following coordinates: anteroposterior, +0.5 mm; mediolateral, +2.3 mm; and dorsoventral, −2.8 mm, relative to the bregma using a flat skull position. We aspirated cortex with a 27–30 G needle to a depth of approximately 2 mm (just past the corpus callosum) and then fit the lens in snugly. We next applied C&B Metabond (Parkell) to cement the lens into place together with a head bar for restraining the mouse when necessary. After the lens implantation, the mice were housed individually under a reversed light/dark cycle. Approximately 2–3 weeks later, we attached a baseplate to guarantee that the microendoscope will be properly aligned with the implanted GRIN lens. To ensure opacity to light, the Metabond was painted with black nail polish.

### Microendoscopic imaging

Microendoscopes (nVista, Inscopix) sampled an area of approximately 600 × 900 µm (pixel dimension, 1.2 µm) at 15 frames/s. Movies were motion corrected with the Inscopix Data Processing Software suite (IDPS, Inscopix). The constrained nonnegative matrix factorization (CNMF-E) algorithm (Pnevmatikakis et al., 2016; Zhou et al., 2018) was applied to motion-corrected movies to detect putative somata and extract traces of fluorescence changes over time. False-positives in the CNMF-E output (i.e., non-neuronal spatial footprints and fluorescence traces) were filtered out of the final dataset manually. Data from one mice with weak transfection or from sessions from all mice with too few somata were discarded.

To track individual cells across imaging sessions, we used *CellReg*—a well-established probabilistic approach to automatically register cells across multiple imaging sessions in Ca^2+^ imaging data (Sheintuch et al., 2017). Briefly, we used CNMF-E to generate spatial footprints for all detected cells in the relevant imaging sessions. Footprints from the earliest session served as a reference map. Footprints from subsequent sessions were aligned to this reference by correcting for rotational and translational differences. The algorithm yields a list of cells, each representing the same cell over multiple sessions. For each set of cells representing the same cell in different imaging sessions, the probability that they all belong to the same cell was calculated (*p*_same_). We included a cell in our analysis if *p*_same_ > 0.5 and the distance between the centroids across sessions was <14 µm. To quantify the registration confidence for each cell, CellReg provides a strict register score index (ranging between 0 and 1), which factors in the registration certainty of all cell pairs. In all cases, the average register score was >0.97.

For each somatic region of interest (ROI), detected using CNMF-E, an annulus was defined as a ring of pixels with the same area as the somatic ROI, whose inner diameter is the distance of the point on the border of the ROI that is farthest from its center of mass plus five additional pixels. In annular ROIs, we extracted fluorescence changes over time (Δ*F*/*F*) such that $\Delta F/F=\frac{\left(F-F_{0}\right)}{F_{0}}$, where *F* represents the raw fluorescence recorded and F_0_ denotes the minimal averaged fluorescence across 2 s periods (with 1 s long overlaps) throughout the measurement.

### Behavioral paradigm

In each imaging session, mice were head restrained on a running saucer, mounted with a microendoscope and placed in a behavior chamber (21 cm × 18 cm × 13 cm) lit by diffuse infrared light, where they could move freely. Naive mice underwent two free movement imaging sessions, in which their spontaneous movements were monitored. Next, the mice were placed under a food restriction regime, and their weight was maintained at approximately 85% of its initial value throughout training. For 2 h before habituation or training sessions, the mice were also water-deprived. Each mouse underwent three habituation sessions, in which it was required to enter its head into a designated port in the behavior chamber and consume a sucrose–water mixture (30% sucrose). Head entries were detected using an infrared photobeam sensor (Med Associates). The session ended after 30 min or once the mouse successfully consumed the sucrose–water mixture 10 times.

Habituated mice underwent an operant conditioning paradigm in which an auditory conditioned stimulus (CS) was associated with a sucrose–water mixture reward. Each conditioning session consisted of 42 randomly spaced trials (an average of 30 or 60 s between consecutive trials). In each trial, one of two auditory cues (CS+ or CS−; 3,200 and 4,300 Hz, respectively) was presented in a pseudo-random manner for 10 s. If the mouse entered its head into the port while the CS+ was being presented, the reward was delivered. A mouse entering its head into the port during CS− presentation was not rewarded. During conditioning, microendoscopic imaging was performed on alternating imaging sessions. The terms naive, intermediate, and expert sessions refer to the 1st, 7th (9th in one mouse), and 15th (13th in one mouse) training sessions, respectively. Advanced training sessions are sessions 11, 13, and 15. Once the association between the CS+ and the reward was fully formed, the mice underwent an extinction paradigm. Extinction sessions were identical to conditioning sessions, but no reward was delivered. The behavioral paradigm was designed using the Med-PC software suite (Med Associates). The training took place during the dark phase of the reversed light/dark cycle.

### Monitoring and analyzing mouse kinematics

In all imaging sessions, mouse kinematics were monitored using an accelerometer (HARP, Champalimaud Foundation Hardware Platform) and two video cameras (Firefly MV FFMV-03M2 M, FLIR Systems), which were all synchronized with the microendoscope. The accelerometer was mounted on the mouse’s head and recorded the body acceleration in the anterio-posterior (BA_AP_), mediolateral (BA_ML_), and dorsoventral (BA_DV_) axes. As previously described (Klaus et al., 2017), the total body acceleration (BA) was defined as follows:

$$\mathrm{BA}=\sqrt{{\left(BA_{\mathrm{AP}}\right)}^{2}+{\left(BA_{\mathrm{ML}}\right)}^{2}+{\left(BA_{\mathrm{DV}}\right)}^{2}}.$$

**Figure 2.**
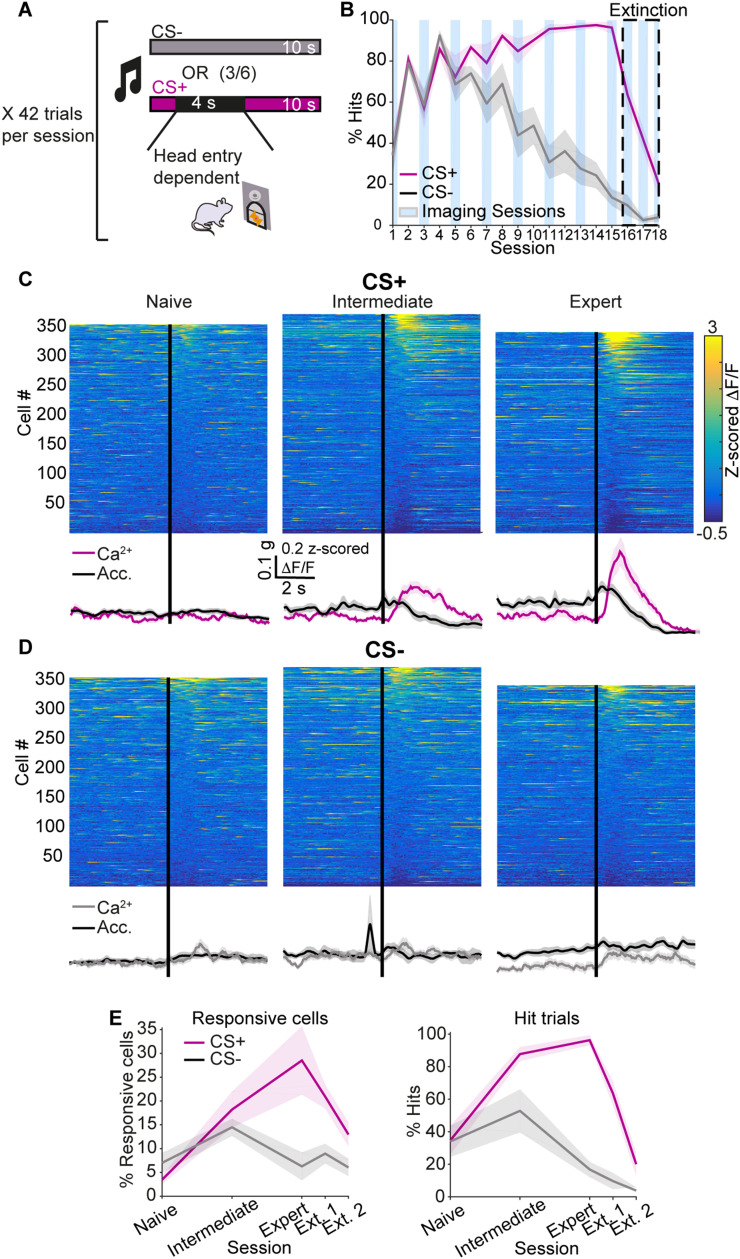
Responses to task-related cues develop with training. ***A***, Mice were trained in an operant conditioning paradigm to associate an auditory CS with a sucrose–water mixture reward. Each session consisted of 42 trails. In each trial, a cue (CS+ or CS−) was presented for 10 s in a pseudo-random manner. If the mouse entered its head into a designated port during the 10 s time window following CS+ (but not CS−) onset, reward was available for 4 s. ***B***, The learning rate quantified as the portion of cue presentations during which the mouse entered its head into the port (% hits). Blue rectangles mark imaging sessions and the dashed box marks extinction sessions. ***C***, Color-coded matrix showing neuronal activity around CS+ presentation averaged across all CS+ presentation events for naive (left), intermediate level (middle) and expert (right) mice. Average Ca^2+^ activity across the population of imaged neurons and average total body acceleration across CS+ presentation events are represented by pink and black traces, respectively (bottom). See Fig. 2-1 for the development of the total body acceleration profile around CS+ presentation as training progresses. ***D***, Same as C, for CS− presentation. Average Ca^2+^ activity across the population is represented by gray traces. ***E***, Rate of responsive neurons (left) and “hit” rate (right, same data as in panel B) around CS+ (pink) and CS− (gray) presentation on various stages of training. Shaded areas represent the S.E.M. g in scale bar is the gravitational constant 9.81 m/s^2^. See Fig. 2-2 for the relationship between the neural responses around CS+ presentation and total body acceleration or reward delivery.

Movement onset and offset times were detected by setting a threshold on the total body acceleration**.** For each mouse and each session, we plotted the distribution of total body acceleration values. The distributions were bimodal, and the threshold was manually set as the middle point between the two peaks (Fig. 1–2*A*,*B*). Frames in which the acceleration trace was above the threshold were considered movement frames, and frames in which the acceleration trace was below the threshold were considered rest frames. The average threshold (mean ± SD) was 0.0584 ± 0.0076 g (the gravitational acceleration). To verify the reliability of movement onset and offset detection, the results for each session were spot-checked against video recordings.

To identify movement onset events accompanied by small or large acceleration changes, we detected the maximal value of the acceleration in a 2 s time window following each movement onset. We then subtracted from the maximal value the value of the acceleration at the time of movement initiation to obtain the amplitude of acceleration change. The amplitudes were divided into quartiles. Movement onsets corresponding to acceleration changes in the 1st quartile were considered small acceleration change movements. Movement onsets with acceleration changes in the 4th quartile were large acceleration change events. The identification of CS+ presentation events accompanied by small or large acceleration changes was conducted similarly.

Ipsi- and contra-lateral turns and grooming events were detected based on movies of mouse behavior recorded using an overhead video camera. We tracked the position of the mouse by applying the DeepLabCut algorithm (Mathis et al., 2018) to video recordings. Results were then used in combination with either a custom-made MATLAB (MathWorks) code to identify times in which the mouse performs right and left turns, or the Janelia Automated Animal Behavior Annotator (JAABA) to identify grooming bouts (Kabra et al., 2013). Only sessions with at least five grooming bouts were included in the analysis.

### Immunohistochemistry

Mice were deeply anesthetized and perfused transcardially with 0.1 M phosphate buffer (PB) followed by ice-cold 4% paraformaldehyde. 50 µm coronal sections of the striatum were incubated overnight at 4°C in CAS-Block (Life Technologies) with a single primary antibody (see Table 1). On the second day, sections were washed in phosphate buffered saline (PBS) and incubated for 3 h at room temperature with the appropriate fluorophore-conjugated species-specific secondary antibody: Cy3 donkey anti-goat (1:1,000, Abcam), Cy3 donkey anti-mouse (1:1,000, Jackson ImmunoResearch Laboratories), Cy3 donkey anti-sheep (1:1,000, Jackson ImmunoResearch Laboratories), or Alexa 647 donkey anti-rabbit (1:1,000, Abcam). Brain sections were rinsed in PBS and directly cover-slipped by a fluorescent mounting medium (VECTASHIELD, Vector Laboratories). Sections were imaged using a laser-scanning confocal microscope (Nikon A1 Plus, Nikon Corporation) using a 20× lens (NA, 0.75).[Table T2]

**Table 1.** Primary antibodies used in this study

Molecular marker	Host animal	Dilution	Source and catalog number	B-cell lineage	Research Resource Identifier (RRID)
Choline acetyltransferase (ChAT)	Goat	1:100	Millipore (AB144P)	Polyclonal	AB_90661
DARPP32	Mouse	1:50	Santa Cruz Biotechnology (SC-271111)	Monoclonal	AB_10610055
Parvalbumin (PV)	Sheep	1:500	R&D Systems (AF 5058)	Polyclonal	AB_2173907
Somatostatin (SOM)	Rabbit	1:100	Peninsula Laboratories (T-4102)	Polyclonal	AB_518613

**Table 2. T2:** Statistical table

	Data structure	Type of test	*p* value
a	Binned correlations	Linear regression	0.03
b	Binned correlations	Linear regression	0.62
c	Binned correlations	Linear regression	0.33
d	Binned correlations	Linear regression	0.03
e	Binned correlations	Linear regression	0.15
f	Binned correlations	Linear regression	0.5
g	Binned correlations	Linear regression	0.9
h	Binned correlations	Linear regression	0.44
i	Binned correlations	Linear regression	0.37
j	Binned correlations	Linear regression	0.98
k	Binned correlations	Linear regression	0.95
l	Binned correlations	Linear regression	0.63
m	Ranks	Two-tailed Wilcoxon signed-rank test for paired replicates	0.03125
n	Ranks	Two-tailed Wilcoxon signed-rank test for paired replicates	0.0625
o	Ranks	Two-tailed Wilcoxon signed-rank test for paired replicates	0.03125
p	Ranks	Two-tailed Wilcoxon signed-rank test for paired replicates	0.125

### Data analysis

Data analysis was conducted using custom-made MATLAB (MathWorks) code. Peak events in Ca^2+^ traces were detected using a MATLAB peak-finding algorithm with the condition that the peak is larger than 2 SD of the Ca^2+^ trace, as well as the absolute value of the largest negative deflection in the signal. Peristimulus time histograms (PSTHs) around various behavioral events were generated based on these detected Ca^2+^ peaks.

Event triggered averages (ETAs) were calculated for individual neurons by considering a 5 or 10 s time window around all behavioral events of a given type (e.g., movement onset, cue presentation, reward delivery) and averaging the Ca^2+^ or acceleration trace across events.

To divide significantly responsive neurons into positively and negatively modulated neurons, we calculated the average ETA during the 1 s time windows immediately before and after event onset. If the average after the event onset was larger than the average before it, the neuron was considered positively modulated. Otherwise, it was considered negatively modulated.

To calculate pairwise correlations during rest, we detected rest events that lasted at least 2.5 s and considered a 2 s time window starting at 250 ms after the movement termination. For each pair of neurons, we calculated the correlations between their Ca^2+^ traces for each of these 2 s time windows and then averaged over the various rest events, resulting in a single correlation value for each pair of neurons. Pairwise correlations around movement onset were calculated the same way, but a 2 s time window centered around movement onset was considered for every movement event.

To test whether pairwise correlations depend on the distance between neurons, we first constructed a frequency plot of the distance between neuronal pairs’ centers (bin size 10 µm). Then, we averaged the correlations between neuronal pairs belonging to the same bin, such that each distance bin was represented by a single average correlation value. A MATLAB function was then used to fit the data with a linear regression model.

To quantify response reliability around movement onset and CS+ presentation, we considered for each event type only neurons whose signals were significantly positively modulated around the onset of this event. For each neuron and each event type, we measured the portion of the behavioral events for which the neuron produced a significant Ca^2+^ transient in a 1 s time window following event onset.

To determine the temporal relationship between somatic and annular signals, we detected peaks in the somatic Ca^2+^ trace and averaged both somatic and annular signals around these times. Signals were first averaged over all of the events in each soma-annulus pair, and the resulting traces were then averaged over the pairs. Decay time constants τ were extracted by fitting a decaying exponent ( a⋅exp(−t/τ)+b, where *t* is time and a and b are constants) to the average signals for each soma-annulus pair. For the purpose of this analysis only, somatic traces were not extracted using CNMF-E, as was done in the rest of the manuscript. For each somatic ROI, the raw fluorescence trace of the corresponding annulus was subtracted. Somatic Δ*F*/*F* traces were then extracted based on the subtracted traces using the approach described above for annular traces.

### Statistics

The nonparametric two-tailed Wilcoxon rank-sum test (RST) was used for nonmatched samples, and the nonparametric Wilcoxon signed-rank test (SRT) was used for matched samples (see Table 2). Shaded areas around curves represent standard errors of the mean.

To determine whether an individual neuron significantly modulated its activity around a given event, a bootstrapping-based strategy was employed (Fig. 1-2). First, we detected instances in which the neuron’s Ca^2+^ ETA crossed a threshold (the 85th percentile). For each such instance, we counted the number of consecutive frames that the ETA spent above the threshold. We next identified the instance corresponding to the largest number of consecutive frames and calculated the area between the ETA and the horizontal line representing the threshold for that instance (Fig. 1-2*D*). To create simulated samples, each neuron’s Ca^2+^ trace was circularly shifted by a random number of frames, and the ETA and area were recalculated around the same event times. This was repeated 2,000 or 5,000 times to generate a null distribution (Fig. 1-2*E*).

Outliers were detected using a MATLAB outlier detection algorithm. A data point was considered an outlier if its value was more than 1.5 interquartile ranges above the upper quartile (75%) or below the lower quartile (25%). Null hypotheses were rejected if the *p*-value was <0.05.

### Joint JPSTH calculation

The JPSTH conveys the temporal dynamics of noise correlation between a pair of simultaneously imaged neurons (Aertsen et al., 1989; Gerstein and Perkel, 1969). Each repetition of the relevant behavioral event (the onset of a spontaneous movement or CS+ presentation) was considered a separate trial. For each pair of neurons, we calculated the raw JPSTH matrix, in which the value in the *t*_1_–*t*_2_-th bin represents the number of trials in which neuron 1 and neuron 2 produced Ca^2+^ transients in time bins *t*_1_ and *t*_2_, respectively.

Cells modulated the rate of their Ca^2+^ transients around the various behavioral events. To account for the correlations due to the covariation in Ca^2+^ signals, the shift predictor matrix was calculated and subtracted from the raw JPSTH (Aertsen et al., 1989). The calculation of the shift predictor matrix was identical to that of the raw JPSTH, except that neuron 2 trials were circularly shifted. Namely, the *n*th trial in neuron 1 was compared to the (*n + m*)th trial in neuron 2, where *m* is the shift. We repeated this for all possible shifts and averaged the resulting matrices to obtain the shift predictor matrix. The corrected JPSTH was then derived by subtracting the shift predictor bin by bin from the raw JPSTH and smoothed using a two-dimensional Gaussian window with a 1 bin SD. The term “JPSTH” refers to the corrected JPSTH. To normalize the JPSTH of a pair of neurons, we divided it by the SDs of their PSTHs (Aertsen et al., 1989; Joshua et al., 2009). The population JPSTH is the average of the normalized corrected JPSTHs of all relevant neuronal pairs. All calculations were performed in 67-ms-wide bins. However, the JPSTH is unitless, and values do not depend on bin width.

To compare the peaks in the diagonal of the population JPSTH to random fluctuations in JPSTH values, we estimated the mean and SD of the diagonal in a 10 s time window far removed (a random shift of least 20 s) from the behavioral events.

### Data and code availability

Any data or code reported in this paper, as well as any other information required to reanalyze the data, are available from the lead contact upon request.

## Results

### Self-initiated movement in freely moving mice strongly modulates the activity of HaR striatal neurons

To examine the response reliability of striatal neurons (presumably to cortico- and thalamostriatal inputs) during naturalistic behaviors, we used GCaMP mice that serendipitously expressed the genetically encoded Ca^2+^ indicator (GECI) GCaMP6f in a sparse population of striatal neurons consisting of a majority of ∼60% SPNs and ∼30% PV interneurons (see Materials and Methods, Discussion, Fig. 1-1)—two striatal populations that are HaR (Koós and Tepper, 2002; Hammond, 2015; Tepper and Koós, 2016) and typically driven to spike by few large synaptic inputs (Johansson and Silberberg, 2020). Seven mice were implanted with a GRIN lens and mounted with microendoscopes to simultaneously visualize multiple sparsely distributed neurons in the dorsal striatum of freely moving mice during innate behavior (Fig. 1*A–C*). The kinematics of the mice were monitored using video cameras, an accelerometer, and tracking software that were synchronized with the Ca^2+^ imaging, enabling us to compare the collective neuronal activity in different behavioral states.

10.1523/ENEURO.0315-23.2023.f1-1 Figure 1-1 Striatal neurons in the sparse GCaMP mouse are a mixture of neuronal types with an SPN majority. **A:** Immunohistochemical analysis of dorsal striatum of sparse GCaMP mice demonstrates that a large portion of GCaMP6f expressing cells express DARPP32. **B:** Same as A for the co-expression of GCaMP6f and parvalbumin (PV). **C:** Same as A for the co-expression of GCaMP6f and somatostatin (SOM). **D:** Same as A for the co-expression of GCaMP6f and ChAT. Scale bar = 100 μm (A-D). **E:** Rates of co-expression of GCaMP6f and markers for various neuronal subtypes in the striatum. 64±2.3% (mean ± S.E.M; range 56-74.9%, N=7 mice) of GCaMP6f-expressing neurons co-express DARPP32, an established SPN marker (Ouimet et al., 1998); 34.64±3.58% (mean ± S.E.M; range 23-45.5%, N=5 mice) co-express PV; and 8.6±0.7% [mean ± S.E.M; range 6.9-10.3%, N=4 mice; one outlier (29%) was excluded from the analysis] co-express SOM;13.9±2% (mean ± S.E.M; range 7-22.2%, N=7 mice) of GCaMP6f-expressing cells exhibited immunoreactivity to ChAT; Importantly, the analysis for the co-expression of SOM and GCaMP6f was conducted on sparse GCaMP mice that were not included in the microendoscopic experiments. **F:** Rates of co-expression of markers for various neuronal subtypes in the striatum and GCaMP6f exhibit that SPN and PV neurons are represented sparsely among the GCaMP6f-expressing neurons. 4.33±0.91% (mean ± S.E.M; range 1.68-8%, N=7 mice) of DARPP32-expressing neurons co-express GCaMP6f; 0.99±0.58% (mean ± S.E.M; range 0-2.67%, N=4 mice) of PV-expressing neurons co-express GCaMP6f; and 14.82±4.75% [mean ± S.E.M; range 5-37.78%, N=8 mice] of ChAT-expressing neurons co-express GCaMP6f; Each data point represents the co-expression rate for a single mouse. Data points with the same color are from the same individual mouse. Black points represent data from sparse GCaMP mice that were not included in the microendoscopic experiments. Outliers are marked by empty circles. Red line is the median. Box edges are 25^th^ and 75^th^ percentile. Whiskers extend to the most extreme data points not considered outliers. Download Figure 1-1, TIF file.

10.1523/ENEURO.0315-23.2023.f1-2 Figure 1-2 Detection of movement onset and offset times and determination of response significance **A:** Distribution of total body acceleration values from a single session in a representative mouse. Red line represents the threshold, manually set as the middle point between the two peaks in the bimodal distribution. g in the scale bar is the gravitational constant 9.81 m/s^2^. **B:** Total body acceleration trace for the same session. Red line represents the threshold. Green bars mark movement times. **C:** Ca^2+^ activity of a representative neuron around a behavioral event for various trials. Red line marks event onset. **D:** Average response of the same neuron. Dashed line represents an 85^th^ percentile threshold. The statistic for significance testing is the area between the average trace and the threshold (green). **E:** Null distribution of area values generated by bootstrapping. The area for which null-hypothesis is rejected is in blue. The empirical area value for the given neuron is marked by the green asterisk. Download Figure 1-2, TIF file.

**Figure 3.**
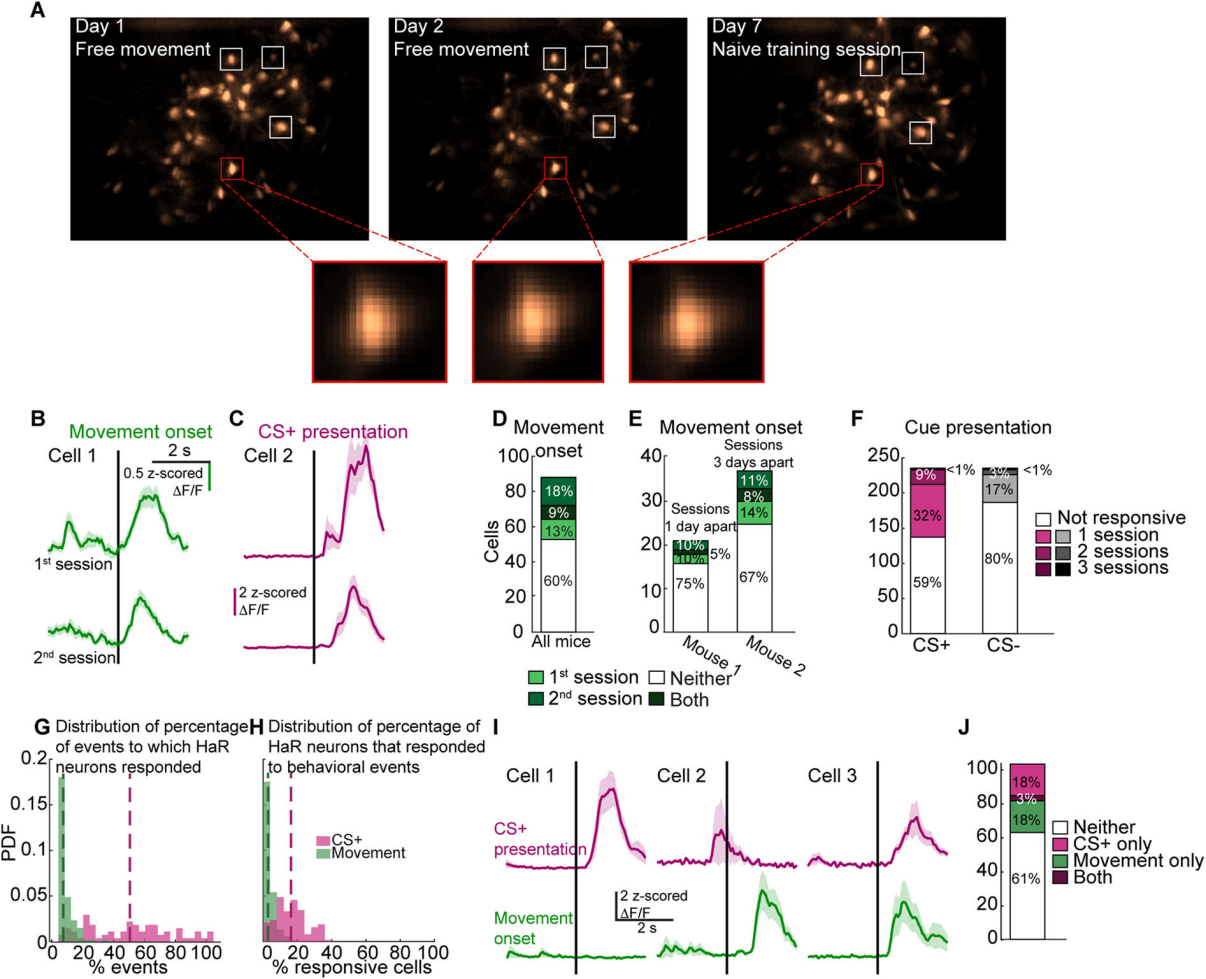
Responses around movement onset and cue presentation are dynamic across imaging sessions. ***A***, Four example cells that were detected on 3 imaging sessions. The images on the bottom represent the footprints produced by CNMF-E of one example cell across the various sessions. ***B***, Average Ca^2+^ signal of an example neuron on the first (top) and second (bottom) free movement imaging sessions that exhibited significant responses (according to our bootstrapping criterion, see Materials and Methods) around movement onset on both sessions. ***C***, Same as A, around CS+ presentation. ***D***, Percentage of neurons significantly responsive on the first (light green) or second (medium green) free movement imaging sessions, both of them (dark green) or neither (white). See Table 3-2 for the time between the two free movement sessions for each mouse. ***E***, Percentage of neurons from mouse 1 (left) and mouse 2 (right) significantly responsive on the first (light green) or second (medium green) free movement imaging sessions, both of them (dark green) or neither (white), that were at most 3 d apart. ***F***, Percent of neurons significantly responsive on one (light), two (medium) or three (dark) advanced conditioning sessions around CS+ (left, pink) and CS− (right, gray) presentation. See Fig. 3-1 for analysis of the reliability around CS+ presentation within a single session. ***G***, PDF of the percent of movement onset (green) and CS+ presentation (pink) events after which the neuron produced a Ca^2+^ event for positively modulated neurons. ***H***, PDF of the percent of neurons that responded with a significant Ca^2+^ transient following each movement onset (green) of CS+ presentation (pink). ***I***, Examples of neurons exhibiting significant and nonsignificant responses around movement onset and CS+ presentation. Cell 1 responded significantly only to CS+ presentation. Cell 2 responded significantly only to movement onset. Cell 3 responded significantly to both. ***J***, Percentage of neurons significantly responsive around movement onset (green), CS+ presentation (pink), both (dark purple) or neither (white) out of the neurons that were detected both in a free movement session and in an advanced conditioning session.

10.1523/ENEURO.0315-23.2023.f1-3 Figure 1-3 Two types of responses around self-initiated movement. **A:** Ca^2+^ activity around movement onset of a representative positively modulated neuron (top). Each trace represents the activity around a single movement initiation event. The average Ca^2+^ activity across the population of positively modulated neurons (middle). Shaded areas represent S.E.M. PSTH of Ca^2+^ events around movement onset in the population of positively modulated neurons (bottom). **B:** Same as A, for neurons that are negatively modulated around movement onset. **C:** The percentage of positively (light) and negatively (dark) modulated neurons, out of the population of neurons significantly modulated around movement onset. ** D:** Rates of neurons positively and negatively modulating their Ca^2+^ signals following movement onset on the first (left) and second (right) free movement imaging sessions. **E:** Rates of imaged neurons positively and negatively modulating their Ca^2+^ signals following movement onset for different significance levels. **F:** Same as C, for neurons significantly modulated around the presentation of task-related cues. **G:** The CDFs of inter-event intervals for positively (light green) and negatively (dark green) modulated neurons during movement. RST. **H:** Same as F, during rest. RST. **I:** CDFs of Ca^2+^ event amplitudes in positively (light green) and negatively (dark green) modulated neurons during rest. RST. **J:** Baseline fluorescence values for positively and negatively modulated neurons. Each data point is the average value across all neurons of the relevant category in the given mouse and session. Empty circles represent outliers. RST. Download Figure 1-3, TIF file.

We began by examining the activity of SPNs and PV interneurons around the onset of self-initiated movement. Six-hundred thirty-seven putative somata were detected in seven mice [two imaging sessions per mouse, 45.5 ± 33.4 (mean ± SD) neurons per session]. Movement onset and offset times were detected by setting a threshold on the total body acceleration of the mouse [see Materials and Methods, Fig. 1-2*A*,*B*, 171 ± 91 (mean ± SD) movements per session]. For each neuron, we calculated the event triggered average (ETA) by averaging the Ca^2+^ signal in a 5 s time window around movement onset across all movement events. We found that the average ETA across the population of imaged neurons increased following movement initiation (Fig. 1*D–F*). The Ca^2+^ signal around the onset of self-initiated movement followed the total body acceleration and peaked approximately 130 ms after the acceleration peak. The PSTH centered around the same events of movement onset exhibited a similar peak, indicating that the population of imaged neurons produced more Ca^2+^ events following movement onset (Fig. 1*E*). Repeating the analysis around movement offset times generated a mirror image of the movement onset responses (Fig. 1*F*), suggesting that neurons modulate their activity when movement is initiated and that, at the population level, the modulation is maintained throughout the movement. To determine whether the activity of an individual neuron was significantly modulated around movement onset, we devised a bootstrapping-based method (see Materials and Methods, Fig. 1-2*C–E*). Twenty seven percent of detected neurons were found to be significantly responsive around the onset of self-initiated movement (Fig. 1*G*). Thus, while movement responsiveness is evident at the population level, only a subset of the imaged neurons consistently modulate their Ca^2+^ signals around movement onset in a given imaging session.

**Figure 4.**
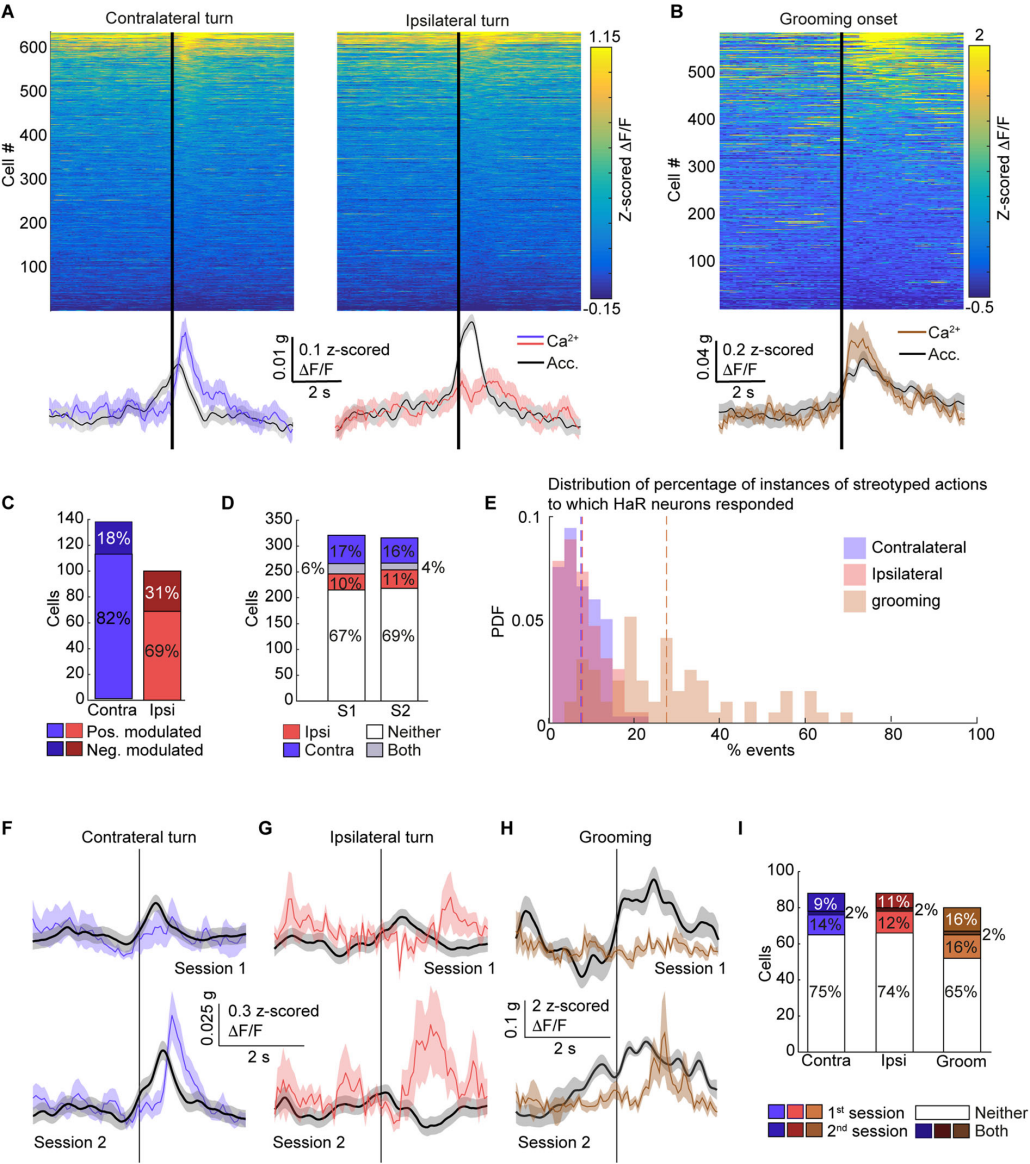
Responses around ipsi- and contralateral turns and around grooming are dynamic across imaging sessions. ***A***, Color-coded matrix showing neuronal activity around the onset of contra- (left) and ipsilateral (right) turns averaged across all turning events for all mice. Average Ca^2+^ activity across the population of imaged neurons is represented by green and purple traces for contra- and ipsilateral turns, respectively. The average total body acceleration across the relevant turning events are represented by black traces (bottom). ***B***, Same as A, around grooming initiation. ***C***, Percentage of positively (light) and negatively (dark) modulated neurons out of the neurons significantly responsive around contra- (green) and ipsilateral (purple) turns on the two free movement sessions. ***D***, Percentage of neurons significantly responsive around ipsilateral turns only (purple), contralateral turns only (green), both turning direction (gray) or neither (white) on the two free movement sessions. ***E***, PDF of the percent of contra- (green) and ipsilateral (purple) turns and grooming initiation (orange) events after which the neuron produced a Ca^2+^ event for positively modulated neurons. ***F***, Average Ca^2+^ signal of an example neuron on the first (top) and second (bottom) free movement imaging sessions that exhibited a significant response around contralateral turns on the second session but not on the first. ***G***, Same as F, for ipsilateral turns. ***H***, Same as F, for grooming initiation. ***I***, Percentage of neurons significantly responsive around contralateral turns (green), ipsilateral turns (purple) and grooming initiations (orange) on the first (light) or second (medium) free movement session only, both (dark) or neither (white) out of the neurons that were detected on both sessions. g in scale bar is the gravitational constant 9.81 m/s^2^.

A closer examination of the ETAs of individual significantly responsive neurons around the onset of self-initiated movement revealed that they exhibit two types of responses (Fig. 1*E,F* and Fig. 1-3): while a majority of responsive neurons (78%) increase their rate of Ca^2+^ transients as the total acceleration of the mouse increases (positively modulated, top part of matrix in Fig. 1*E*- and Fig. 1-3*A*), some significantly responsive neurons (22%) produce less Ca^2+^ transients when the mouse is moving (negatively modulated, bottom part of matrix in Fig. 1*E* and Fig. 1-3*B*). Additionally, movement responses of the imaged neurons were lateralized, displaying a strong preference to contralateral turns over ipsilateral ones (Fig. 4*A,C,D*). These properties are consistent with known activity patterns of SPNs and PV interneurons during spontaneous movement (Albin et al., 1989; Alexander and Crutcher, 1990; DeLong, 1990; Gerfen et al., 1990; Mink, 1996; Hikosaka et al., 2000; Chan et al., 2006; Brown, 2007; Nambu, 2008; Kravitz et al., 2010; Gittis et al., 2011; Cui et al., 2013; Isomura et al., 2013; Jin et al., 2014; Tecuapetla et al., 2014; Perk et al., 2015; Barbera et al., 2016; Klaus et al., 2017; Parker et al., 2018; Gritton et al., 2019).

### Portion of HaR striatal neurons responsive to task-related cues increases systematically as training progresses

To evaluate the response reliability of SPNs and PV interneurons during learning, we trained six mice in an operant conditioning paradigm to associate an auditory CS with a reward. Each training session consisted of 42 trials. In each trial, an auditory cue (CS+ or CS−) was presented for 10 s in a pseudo-random manner. To receive a sucrose–water mixture reward, the freely moving mouse was required to enter its head into the reward delivery port while the CS+ was being presented. A head entry during CS− presentation was not rewarded (Fig. 2*A*).

10.1523/ENEURO.0315-23.2023.f2-1 Figure 2-1 Mice acceleration profiles following CS+ presentation become more stereotyped as training progresses. **A:** Example consecutive total body acceleration traces from a representative mouse around CS+ presentation on naïve training sessions. Each trace represents a single trial. **B:** Same as A, for intermediate training sessions in the same mouse. **C:** Same as A, for expert training sessions in the same mouse. **D:** Box plot of the correlations between the total body acceleration around CS+ presentation in each trial and the average correlation trace across all trials in the same session for naïve, intermediate and expert training sessions. Data from 6 mice are pooled together for each training stage. Outliers are marked by empty circles. The middle line in each box is the median. Box edges are 25^th^ and 75^th^ percentile. Whiskers extend to the most extreme data points not considered outliers. RST. g in the scale bar is the gravitational constant 9.81 m/s^2^. Download Figure 2-1, TIF file.

10.1523/ENEURO.0315-23.2023.f2-2 Figure 2-2 Responses to cue presentation are not determined by total body acceleration or reward delivery. **A:** Average Ca^2+^ activity across the neuronal population (pink) and total body acceleration (black) around CS+ presentations with small (left, 1^st^ quartile) and large (right, 4^th^ quartile) acceleration changes. **B:** Same as A, around movement initiations. Ca^2+^ activity shown in green. Total body acceleration shown in black. **C:** Average Ca^2+^ activity across the population for unrewarded CS+ presentations on various stages of conditioning (light to dark pink), and for the first 10 CS+ presentations on the first extinction session (black). Shaded areas represent S.E.M. g in the scale bar is the gravitational constant 9.81 m/s^2^. Download Figure 2-2, TIF file.

Mice successfully acquired the association between the CS+ and the sucrose–water mixture reward (Fig. 2*B*). To quantify learning, we measured for each cue (CS+ and CS−) the portion of cue presentations during which the mouse entered its head into the port attempting to obtain the reward (“hits”). On advanced training days, mice perform head entries upon almost every CS+ presentation, while the rate of CS− “hits” decreases to ∼15%, indicating that the CS+ was associated with the sucrose–water mixture reward while the CS− was not. Once the association was established, the mice underwent three extinction sessions. Those sessions were identical to conditioning sessions, but no reward was delivered. The association between the CS+ and the reward was quickly extinguished, and mice ceased to perform head entries (Fig. 2*B*).

To characterize the neural activity in various stages of learning, we compared ETAs in a 10 s time window around cue presentation in naive, intermediate level and expert mice (see Materials and Methods). We considered one session per mouse at each learning stage and detected a total of 355, 342 and 373 putative somata on naive, intermediate, and expert training sessions, respectively. In naive mice, the average Ca^2+^ response following CS+ presentation was weak. The population response developed as training progressed and was strong on advanced conditioning sessions when mice were experts at the task (Fig. 2*C*). In contrast, average CS− responses remained weak throughout training (Fig. 2*D*). This was evident in the average ETAs representing the population signal, as well as in the ETAs of individual neurons.

The mice changed their behavior as training progressed. Specifically, as the association between the CS+ and the reward strengthened, mice were quicker to move towards the reward port when the CS+ was presented, resulting in more stereotypical acceleration profiles across CS+ presentations (Fig. 2-1), as well as a larger average acceleration change around CS+ onset (Fig. 2*C*, compare the amplitude of the black trace as sessions progress from naive to expert). This raises the concern that the CS+ responses that we observed are in fact responses to the initiation of the accompanying movement. To address this issue, we divided CS+ presentations on the final conditioning session into two groups according to the size of the associated change in total body acceleration (see Materials and Methods). The average acceleration profiles were very different for the large (4th quartile) and small (1st quartile) acceleration change event groups, and even exhibited opposite change directions (Fig. 2-2*A*). However, the shape and amplitude of the Ca^2+^ responses were very similar in the two cases. In contrast, when we repeated the analysis for movement initiations on free movement sessions, the average Ca^2+^ trace followed the average acceleration trace, with a substantially smaller average Ca^2+^ amplitude for small acceleration change movements compared to large acceleration change movements (Fig. 2-2*B*). Thus, while neuronal responses following CS+ presentation may be affected by the mouse’s movement, these results indicate that they are not determined by the total body acceleration. Finally, CS+ responses continue to appear and increase with training on unrewarded trials and are therefore not dependent on reward delivery (Fig. 2-2*C*).

The event rates of neurons significantly responsive to CS+ or CS− tightly corresponded to task performance (Fig. 2*E*). In naive mice, very few neurons responded around either cue. In intermediate level mice, towards the middle of training, there was an increase in the portion of significantly responsive neurons for both CS+ and CS− presentations. On advanced conditioning sessions, when mice were experts at the task, a larger portion of detected neurons significantly modulated their activity following CS+ presentation, while very few responded to CS− presentation. This profile echoed the progression of learning as represented by the rates of CS+ and CS− “hits”, suggesting that the activity of these neurons is meaningful in the context of the task. Notably, while both the average population signal and rate of significantly responsive neurons increase systematically as training progresses, even in expert mice, only a subset (∼30%) of detected neurons significantly modulated their activity in response to CS+ presentation.

Similarly to movement-responsive neurons, neurons that significantly modulated their signals around task-related cues could be divided into two functional groups—positively and negatively modulated neurons (Fig. 2*C,D* and Fig. 1-3*F*). Here too, the majority of responsive neurons increased the rate of their Ca^2+^ transients following cue presentation, while the rest exhibited less Ca^2+^ events. Thus, subsets of SPNs and PV interneurons modulate their activity around self-initiated movements as well as task-related cues in an operant conditioning paradigm.

### Individual HaR striatal neurons are recruited to respond to the same behavioral events in a nonreliable fashion across and within imaging sessions

We next examined the reliability of neuronal responses around various behavioral events. Will a neuron that is significantly responsive around a specific behavior in a given imaging session continue to code for it on subsequent sessions? Our experimental approach allowed us to track individual neurons across multiple imaging sessions (Fig. 3*A*, see Materials and Methods). To examine the stability of movement responses, we detected neurons that were active on two free movement sessions. Out of 88 such neurons, 22% significantly responded around movement onset on the first session, 27% responded on the second session, and only 9% significantly modulated their signals around self-initiated movement on both free movement sessions (Fig. 3*D*). While the 9% overlap is greater than the expected rate if neurons are randomly recruited to respond around movement onset (e.g., 6%), the identity of significantly responsive neurons appears to be highly dynamic across imaging sessions.

10.1523/ENEURO.0315-23.2023.f3-1 Figure 3-1 HaR neurons are dynamically recruited within a single session and around a consistent behavior. **A:** Average Ca^2+^ activity for an example neuron around CS+ presentation across all trials (black), odd trials only (light pink) and even trials only (dark pink). Shaded areas represent S.E.M. Asterisks mark significant responses with black, light pink and dark pink corresponding to all, odd and even trials, respectively. **B-C.** Same as A, for different neurons. **D.** Venn diagram of significantly responsive neurons when bootstrapping-based significance testing was performed using all trials (black), odd trials only (light pink) or even trials only (dark pink). **E.** Same as D, for all trials (black), the first (light pink) and the last half of trials (dark pink). Download Figure 3-1, TIF file.

10.1523/ENEURO.0315-23.2023.t3-2 Table 3-2 Time between the two free movement sessions for the various mice. Download Table 3-2, DOC file.

The two free movement sessions in each mouse were between 1 and 29 days apart (see Table 3-2). To test if a large interim could account for the high variability in the populations of responsive neurons, we focused on two mice whose free movement sessions were 1 and 3 days apart (mice 5 and 6 in Table 3-2, Fig. 3*E*). This analysis revealed very similar percent overlap between the populations of neurons significantly responsive on the two sessions, suggesting that the low reliability is not due to changes occurring slowly over time.

To assess the reliability of the responses around task-related cues, we examined the signals of neurons that were detected on three advanced training sessions (2–4 d apart) around the presentation of the CS+ and CS– (Fig. 3*F*). Of the 233 detected neurons, 32% significantly responded to CS+ presentation on a single session, 9% were responsive on two sessions, and only one neuron significantly modulated its signal following CS+ presentation on all three sessions. Similarly, 17% of the neurons were significantly responsive around CS− presentation on a single session, 3% were responsive on two sessions, and one neuron was responsive on all three sessions. These percentages of overlap are consistent with neurons being randomly and independently recruited to respond around cue presentation on different imaging sessions.

An important caveat is that the movement events that we considered, detected by setting a threshold on the total body acceleration, can include a wide range of movements. Thus, the changes in the identity of significantly responsive neurons between the first and second free movement sessions could be due to differences in the behavior of the mice (Liberti et al., 2022). To address this concern, we analyzed video recordings of mouse behavior to detect grooming initiations and turning events in the ipsi- or contralateral direction (see Materials and Methods, Fig. 4). Repeating the analysis for these more consistent, strictly defined movements, we found again that the rate of neurons significantly responsive around a given movement type on both free movement sessions was most consistent with random recruitment (Fig. 4*I*).

This result is further supported by the dynamic identity of CS+ responsive neurons over advanced training days. As mice become experts in the task, the acceleration profile of their behavior around task-related cues becomes much more consistent (Fig. 2-1). Our analysis of CS+ and CS− responses across advanced imaging sessions indicates that while the percentage of significantly responsive neurons increases with training, their identity is dynamic and shifts from session to session (Fig. 3*F*). Thus, while behavioral variability may still contribute to the shifting identities of significantly responsive cells, our results suggest that it is not the sole cause.

To examine the reliability of the responses within a given session, we considered significantly responsive neurons whose signals were positively modulated around movement onset. Positively modulated neurons are the majority of significantly responsive neurons and tend to generate more Ca^2+^ events following movement onset compared to prior to it (Fig. 1-3). For every such neuron, we calculated the portion of movement initiations after which it produced a Ca^2+^ event. Interestingly, even neurons that were categorized as positively modulated for a given imaging session only produced Ca^2+^ events in an average of 7.2% of movements (Fig. 3*G*). Repeating the analysis for neurons that were positively modulated around CS+ presentation (Fig. 2*D* and Fig. 1-3*F*) produced a higher percent overlap—neurons responded with a Ca^2+^ event for an average of 46.4% of CS+ presentations, with some cells responsive following 100% of cue presentations. The higher reliability of the responses following CS+ presentation compared to movement onset may reflect a difference in the organization of striatal neurons around spontaneous movement and learned behaviors. Alternatively, the disparity may be due to the lower number of cue presentation events (23 cue presentations per session compared to an average of 171 movement initiation events), as well as the more homogeneous behavior associated with these events (Fig. 2-1). Repeating this analysis for grooming, ipsi- and contralateral turns produced very similar results (Fig. 4*E*), indicating that reliability within a training session is highly dynamic even for more stereotyped behaviors.

Next, for each spontaneous movement initiation and CS+ presentation, we quantified the percentage of HaR neurons that produced a Ca^2+^ transient in a 1.5 s time window following the onset of the behavioral event (Fig. 3*H*). We found that an average of 2.8 and 16.2% of the imaged neurons were active following movement onset and CS+ presentation, respectively, suggesting that a small portion of the HaR population is sufficient to encode each repetition of the behavior. Furthermore, our data indicate that the specific neurons responsive to each repetition of a behavioral event can vary (Fig. 3*D–G*), but the percentage of responsive neurons remains relatively consistent (0–22.7% for movement onset and 0–39.3% for CS+ presentation).

Finally, to further test the hypothesis that HaR neurons are dynamically recruited even during the same behavior, we focused on the expert session, where the acceleration profiles of CS+ triggered movements are more consistent (Fig. 2-1). We split the session into two, considering odd and even trials separately, and tested whether the same neurons respond around cue presentation. Sixty-five and 80 neurons were categorized as significantly responsive based on odd and even trials, respectively. Of these, 44 cells were identified as significantly responsive for both trial subsets (Fig. 3-1*D*). Results were similar when the first or last halves of the trials were analyzed separately, with 92 and 56 neurons significantly responsive based on the first and last half of trials, respectively, and 37 responding significantly in both cases (Fig. 3-1*E*). Thus, while the identity of neurons recruited to respond around CS+ presentation within a session is more consistent than recruitment across sessions (Fig. 3), even within a single session and with relatively consistent behavior (Fig. 2-1), the identity of responsive striatal HaR cells is not constant.

Our results suggest overall low reliability in the responses of SPNs and PV interneurons, in line with our main hypothesis. We observe this inconsistency in the identity of responsive neurons around both the initiation of spontaneous movement and CS+ presentation, suggesting that neural responses around innate and learned behaviors are equally unreliable, contrary to our predictions.

### Individual HaR neurons are recruited to respond to diverse actions in an independent fashion

Imaged neurons significantly modulate their activity following the onset of self-initiated movement and task-related cues (Figs. 1, 2). Do the same neurons respond to the different behavioral events, or are there distinct subpopulations designated to code for each behavior? To address this question, we tracked individual neurons across multiple imaging sessions and examined their responsiveness around movement initiation and CS+ presentation.

One hundred and three neurons were detected on both a free movement session and an advanced conditioning session. Of these neurons, 21% exhibited significant responses around movement onset only, 21% significantly modulated their activity following CS+ presentation only, and 3% were responsive around both movement initiation and cue presentation (Fig. 3*I*). The percent of neurons significantly modulating their signals around both behavioral events is consistent with neurons being randomly and independently recruited to respond to movement initiation and CS+ presentation.

Taken together, our results indicate that while HaR SPNs and PV interneurons consistently modulate their activity around self-initiated movement and CS+ presentation on the population level, the identity of responsive neurons is not maintained. Rather, neurons are dynamically recruited to code for these different behavioral events on separate occurrences.

### Signal correlations between pairs of HaR neurons are independent of distance

Are responsive neurons selected at random with each repetition of the behavior? Or is there a more complex internal organization that our analysis is unable to capture, perhaps due to the vague definition of the behavior we are examining (e.g., movement initiations)? Recent studies have shown that SPNs form spatially compact neural clusters in which nearby neurons are co-active and code for the same type of movement (Barbera et al., 2016; Parker et al., 2018; Shin et al., 2020). Notably, this influential conclusion was drawn from the observation that the correlations between pairs of SPNs decay with distance. Thus, to test whether the unreliability of individual responses in striatal HaR neurons is compensated for by co-activation, we calculated pairwise signal correlations among our imaged neurons and the dependence of signal correlations on the distance between the neurons’ centers.

**Figure 5.**
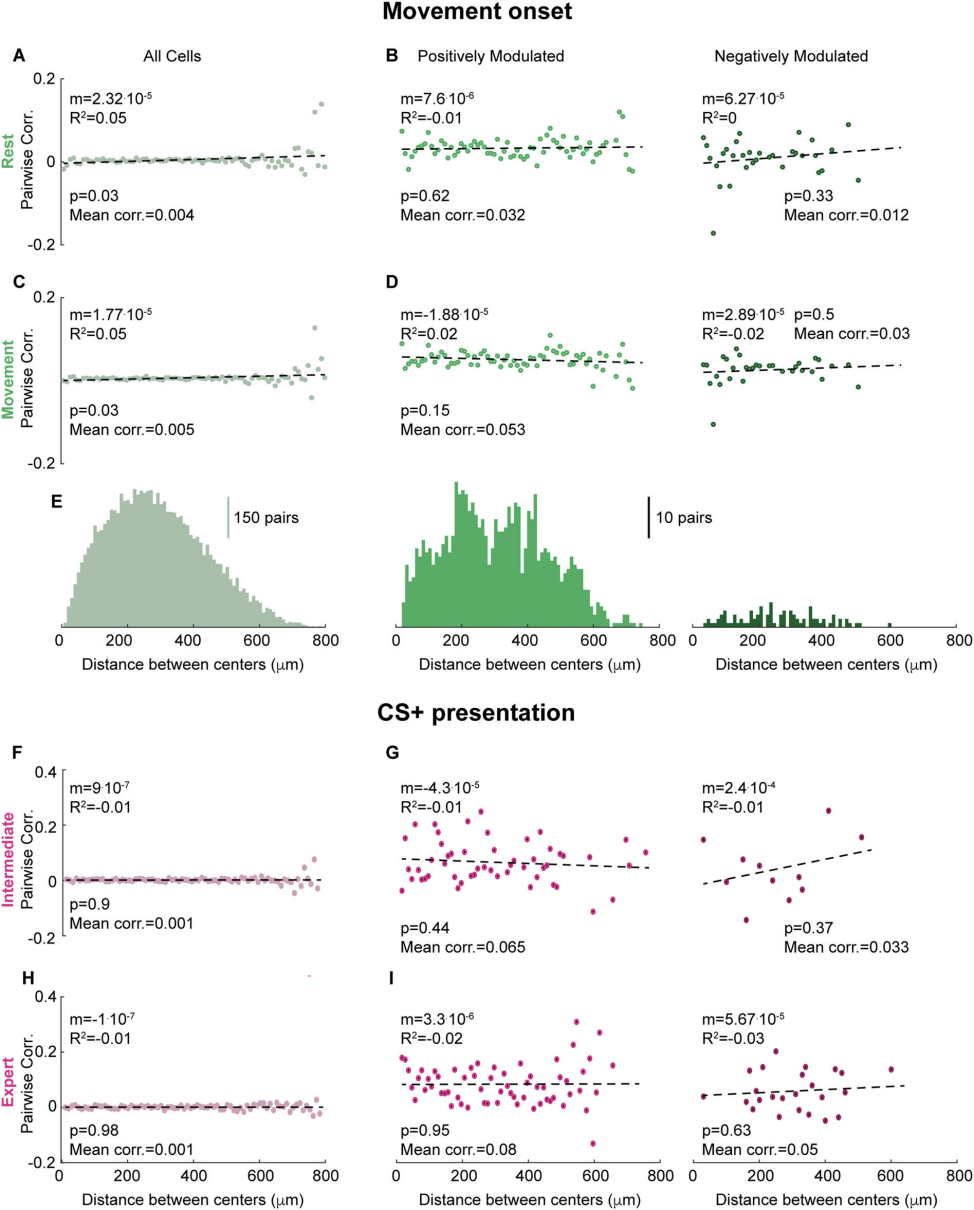
Pairwise correlations are not distance dependent. ***A***, Pairwise correlations as a function of the distance between the neurons’ centers for all pairs of co-imaged neurons during rest. Each point is the average correlation across all neuronal pairs belonging to the relevant distance bin. Dashed line is the linear fit to the data points. ***B***, Same as A, divided into positively (left) and negatively (right) modulated pairs. ***C***, Same as (***A***), around movement onset. ***D***, Same as (***B***), during movement. ***E***, Distribution of neuronal pairs for the various distances for all pairs (left), positively (middle) and negatively modulated pairs (right). Bin width is 10 µm. ***F***, Same as (***B***), around CS+ presentation on intermediate training session. ***G***, Same as (***F***), divided into positively (left) and negatively (right) modulated pairs. ***H***, Same as (***F***), on expert training session. ***I***, Same as (***G***), on expert training session. See Fig. 5-1 for analysis of the correlations in the microendoscopy background neuropil signal and their dependence on distance.

To test whether nearby neurons tend to exhibit similar activity patterns, we calculated signal correlations between the Ca^2+^ traces of pairs of neurons during rest and movement. When all simultaneously imaged neuronal pairs were included, the average correlation (averaged over 10 µm distance bins and then across bins, see Materials and Methods) is very low during both rest (0.004, Fig. 5*A*) and movement (0.005, Fig. 5*C*). When positively and negatively modulated neurons are considered separately, pairwise correlations during both movement states are larger by an order of magnitude (but still weak, 0.03–0.05) and slightly (but not significantly) larger during movement compared to during rest (Fig. 5*B*,*D*). Notably, all of these average values are considerably lower than what was previously reported (Barbera et al., 2016; Shin et al., 2020) (see Discussion). Strikingly, for all functional groups and both movement states, pairwise correlations did not depend on the distance between the neurons’ centers (Fig. 5*A–D*; all cells during rest: mean = 0.004, *m* = 2.32^.^10^−5^, *R*^2 ^= 0.05, *p* = 0.03^a^; all cells during movement: mean = 0.005, *m* = 1.77^.^10^−5^, *R*^2 ^= 0.05, *p* = 0.03^b^; positively modulated during rest: mean = 0.032, *m* = 7.6^.^10^−6^, *R*^2^ = −0.01, *p* = 0.62^c^; negatively modulated during rest: mean = 0.012, *m* = 6.27^.^10^−5^, *R*^2 ^= 0, *p* = 0.33^d^; positively modulated during movement: mean = 0.053, *m* = 1.88^.^10^−5^, *R*^2 ^= 0.02, *p* = 0.15^e^; negatively modulated during movement: mean = 0.03, *m* = 2.89^.^10^−5^, *R*^2^ = −0.02, *p* = 0.5^f^; *n *= 80 distance bins, linear regression).

10.1523/ENEURO.0315-23.2023.f5-1 Figure 5-1 Neuropil signal is highly correlated in space and displays different kinetics then the somatic signals. **A.** Illustration of the sampling of a soma and the surrounding annular region of interest (red). **B.** Pairwise correlations between annular signals as a function of the distance between neuronal centers for all pairs of co-imaged neurons. Each point is the average correlation across all neuronal pairs belonging to the relevant 10 μm distance bin. **C.** Color-coded matrix of the fluctuations in fluorescence as a function of time in 80 somata detected in a single free movement session in a single mouse, with each row representing an individual soma**. D.** Spatial average of signals from all annuli presented in D. **E.** Same as B, with each row representing the signal of the corresponding annulus. **F.** Ca^2+^ signals from a soma-annulus pair. **G.** Distribution of pairwise correlation coefficients for 21,427 simultaneously imaged soma-soma and annulus-annulus pairs. Stars mark the means (0.023 and 0.96 for somata and annuli, respectively). **I.** Average Ca^2+^ signal from the soma and its corresponding annulus averaged over 429 soma-annulus pairs triggered on the somatic Ca^2+^ events. Shaded areas mark S.E.M. **H.** Boxplot of decay time constants for somatic and annular Ca^2+^ signals. The bold line is the median and the whiskers are the 25^th^ and 75^th^ percentiles. Dots represent outliers. Download Figure 5-1, TIF file.

Next, we calculated the signal correlations between the Ca^2+^ traces of neuronal pairs around CS+ presentation. Similarly to movement responses, when all pairs were included, pairwise correlations around cue presentation were very low on both intermediate and expert training sessions (0.001, Fig. 5*F*,*H*). When positively and negatively modulated neurons were considered separately, pairwise correlations were again increased by an order of magnitude, but still weak. Importantly, for CS+ responses, at both stages of training and for all functional groups, pairwise correlations did not significantly depend on the distance between the neurons, either. To conclude, we observe weak signal correlations between pairs of our imaged neurons that are difficult to reconcile with the idea of co-activated clusters of nearby neurons. These correlations are also distance independent for both self-initiated movement and CS+ presentation (Fig. 5*F*–*I*; all cells intermediate: mean = 0.001, *m* = 9^.^10^−7^, *R*^2^ = −0.01, *p* = 0.9^g^; all cells expert: mean = 0.001, *m* = −1^.^10^−7^, *R*^2^ = −0.01, *p* = 0.98^h^; positively modulated intermediate: mean = 0.065, *m* = −4.3^.^10^−5^, *R*^2^ = −0.01, *p* = 0.44^i^; negatively modulated intermediate: mean = 0.033, *m* = 2.4^.^10^−4^, *R*^2^ = −0.01, *p* = 0.37^j^; positively modulated expert: mean = 0.08, *m* = 3.3^.^10^−6^, *R*^2^ = −0.02, *p* = 0.95^k^; negatively modulated expert: mean = 0.05, *m* = 5.67^.^10^−5^, *R*^2^ = −0.03, *p* = 0.63^l^; *n *= 80 distance bins, linear regression), in contrast to previous findings of spatially compact SPN clusters (Barbera et al., 2016; Shin et al., 2020). Interestingly, correlations in the background neuropil activity, represented by the Ca^2+^ signals in the annuli surrounding the various somata (see Materials and Methods), did exhibit a clear distance dependence with pairwise correlations decreasing sharply between 0 and 200 µm (Fig. 5-1B, see Discussion).

### Trial-by-trial covariation among HaR neurons around movement onset are attributable to variability in behavior

Signal correlations between neuronal pairs reflect the degree to which their average responses are modulated together by the sensory or behavioral event. However, signal correlations do not capture the role of the ongoing moment-by-moment synergy between individual neurons. To investigate the propensity of the imaged neurons to be co-active beyond common increases or decreases in the average rate of Ca^2+^ transients, we calculated joint JPSTHs for the various neuronal pairs (Gerstein and Perkel, 1969; Aertsen et al., 1989; Joshua et al., 2009). The JPSTH is a measure of noise correlations or the degree to which trial-by-trial fluctuations in the response are shared by a pair of neurons. It is derived by subtracting the shift predictor matrix from the product of the PSTHs of the two neurons (Aertsen et al., 1989) (see Materials and Methods), resulting in an estimate of unpredicted correlations.

**Figure 6.**
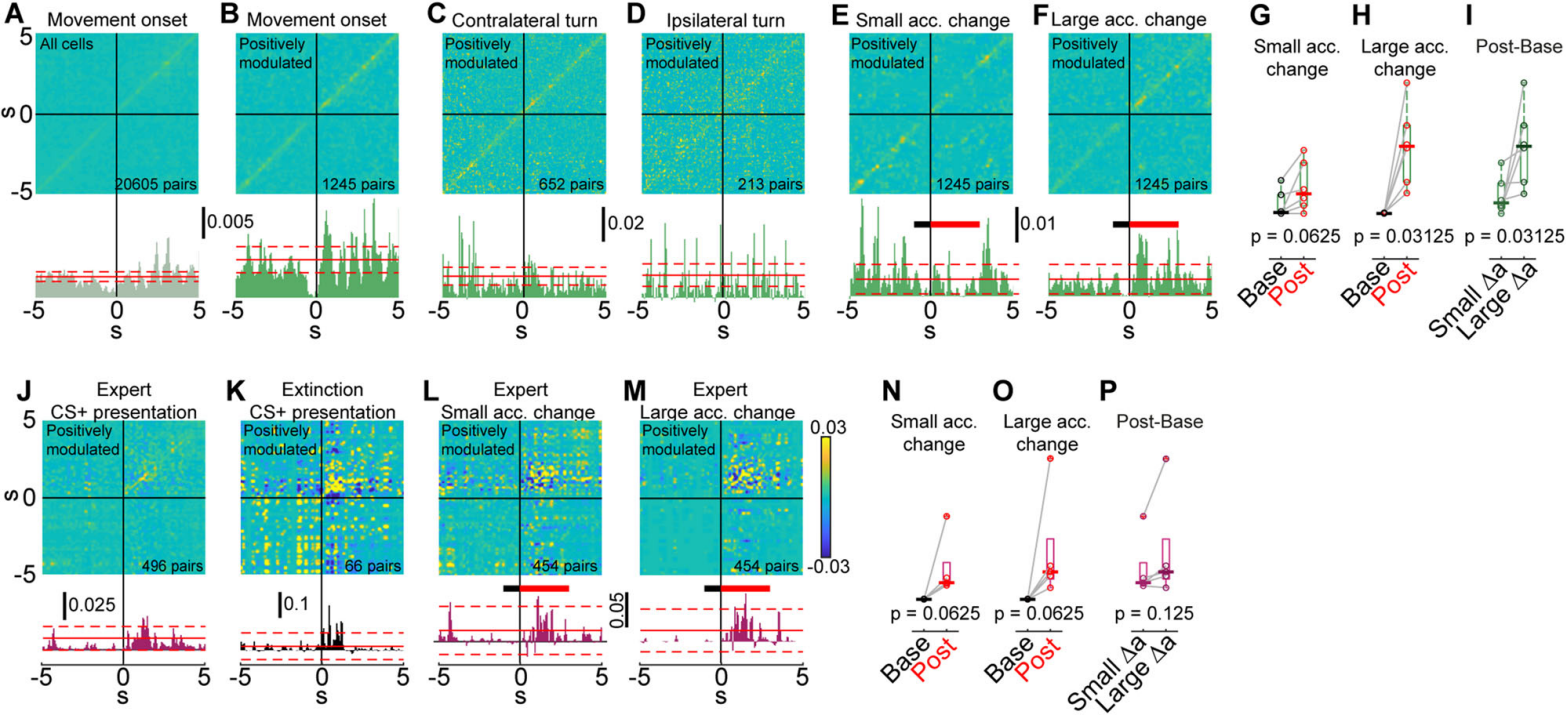
Behavioral variability more strongly affects trial-by-trial correlations around spontaneous movement than during learned behaviors. ***A***, Population joint JPSTH, averaged across all neuronal pairs, centered around movement onset (top). Bar plot shows values of the JPSTH diagonal (bottom). Solid red line represents the mean value of the JPSTH diagonal in a 10 s time window far removed from movement onset events. Dashed red lines represent the mean ± the SD. ***B***, Same as (***A***), for pairs of positively modulated neurons. ***C***, Same as (***B***), for contralateral turns. ***D***, Same as (***B***), for ipsilateral turns. ***E***, Same as (***B***), for movement onset events with a small acceleration change. Thick lines mark the time windows for calculation of baseline (black) and post-event (red) maximal values. ***F***, Same as (***B***), for movement onset events with a small acceleration change. (***G***) Boxplot of baseline (black) and post-event (red) maximal values of population JPSTH diagonals around movement onset for small acceleration change movements. Each circle represents one mouse (*N* = 6 mice). ***H***, Same as (***G***), for large acceleration change movements. ***I*** Boxplot of the difference between the maximal value of the population JPSTH diagonal in the post-event and baseline time windows for movement onset with small (left) and large (right) acceleration changes. The bold line is the median and the whiskers are the 25th and 75th percentiles. Red crosses represent outliers. ***J***, Same as (***B***), for CS+ presentations on expert training session (top). ***K***, Same as (***J***), for first 10 trials of the first extinction session. ***L***, Same as (***E***), for CS+ presentations. ***M***, Same as (***F***), for CS+ presentations. ***N***, Same as (***G***), for CS+ presentations (*N* = 5 mice). ***O***, Same as (***H***), for CS+ presentations. ***P***, Same as (***I***), for CS+ presentations. The bold line is the median and the whiskers are the 25th and 75th percentiles. Red crosses represent outliers.

To assess noise correlations in the responses to self-initiated movement, we calculated the JPSTHs for pairs of neurons around movement onset. The JPSTHs of all neuronal pairs were then normalized and averaged to derive the population JPSTH (Aertsen et al., 1989; Joshua et al., 2009) (see Materials and Methods). The diagonal of the resulting matrix quantifies the average time-varying modulation of zero-lag noise correlation across the population (Fig. 6*A*). Noise correlation values were similar along the entire diagonal, except for the 1 s time window immediately before movement initiation showing a correlation drop (Fig. 6*A*). This reduction is likely due to the decreased acceleration and neuronal activity that mark the period leading up to movement onset (Fig. 1*E*).

Next, the analysis was repeated, considering only pairs of positively modulated neurons. Focusing on this population resulted in a larger peak in noise correlation immediately following movement initiation (Fig. 6*B*). The diagonals of the population JPSTHs for pairs of negatively modulated neurons or pairs with opposite modulation directions did not exhibit significant peaks (data not shown).

Movement onset times were detected by setting a threshold on the total body acceleration of the mouse and therefore include a wide range of movements. To test whether joint trial-by-trial variations in neuronal activity arise from variability in the mouse’s behavior, we repeated the analysis for subsets of more stereotyped movement events. Limiting the analysis to contra- or ipsilateral turns eliminated the peak in the diagonal of the population JPSTH (Fig. 6*C,D*). Finally, the same calculations were conducted focusing separately on movements with small (1st quartile) and large (4th quartile) acceleration changes (Fig. 6*E*,*F*). While the JPSTH diagonal for movements accompanied by a small acceleration change exhibits a small peak, which does not exceed random fluctuations in the values of the diagonal, movements with large acceleration changes induce a stronger peak in noise correlation.

To examine the significance of this effect, we evaluated the peak values of the diagonal of the population JPSTH for each mouse. Specifically, we compared the peak values within a 0.5 s time window preceding movement onset (baseline) to those within a 1.5 s window immediately following movement onset (post). Our analysis indicated that while there was a significant difference in peak values between the baseline and post-event periods for movements with large acceleration changes (Fig. 6*H*; *p* = 0.03125^m^, SRT, *n *= 6 mice), this difference was not significant for movements with small acceleration changes (Fig. 6*G*; *p* = 0.0625^n^, SRT, *n *= 6 mice). When we subtracted baseline peaks from post-event peaks and compared these values for small and large acceleration change movement onsets, we found that the difference was significant (Fig. 6*I*; *p* = 0.03125^o^, SRT, *n *= 6 mice). Taken together, these results suggest that trial-by-trial correlations around the onset of self-initiated movements are mostly due to variability in the movement of the mouse driving a temporal covariation among neurons across repeated events.

### Trial-by-trial correlations following the presentation of task-related cues

To investigate trial-by-trial correlations during learning, we calculated the JPSTHs of neuronal pairs around CS+ presentation focusing on the expert training session and the first extinction session. When all neuronal pairs were included, the diagonals of the population JPSTHs exhibited no peaks for any stage of training (data not shown). Similarly to the movement onset JPSTH, focusing on pairs of positively modulated neurons resulted in substantial noise correlation peaks following CS+ presentation in expert mice (Fig. 6*J*). Importantly, mouse behavior following CS+ presentation, particularly at advanced stages of training, is relatively uniform. First, mice successfully obtain the sugar-water reward in the vast majority of trials during the expert training session (128 out of 137 trials in 6 trained mice). Additionally, mice acceleration profiles following CS+ presentation become less variable as training progresses (Fig. 2-1). Next, the analysis was repeated focusing on the first 10 extinction trials. These trials were unrewarded, like the rest of the extinction session, but they occurred early enough in the session so that the association between the cue and the reward had not yet been lost. In this case as well, noise correlations rise shortly after the presentation of the CS+ (Fig. 6*K*).

To further reduce the variability in the behavior, we divided expert session trials into those accompanied by small and large acceleration changes (Fig. 6*L*,*M*). In both cases, the diagonals of the population JPSTHs maintain their peaks following CS+ presentation, with no significant difference in the change in JPSTH diagonal peaks around CS+ presentations with small and large acceleration changes (Fig. 6*P*; *p* = 0.125^p^, SRT, *n *= 6 mice).

In summary, it appears that trial-by-trial correlations around the onset of self-initiated movements arise mostly from variability in the behavior of the mouse. In contrast, noise correlations surrounding the presentation of task-related cues persisted when we examined subsets of behavioral events with more similar characteristics. In particular, our analysis revealed a significant difference in peak JPSTH values for self-initiated movements with small and large acceleration changes (Fig. 6*I*), but the same analysis did not produce a significant difference when applied to CS+ presentations (Fig. 6*P*). Thus, while evidence for dynamic formation of correlated assemblies on individual actions is weak, if it occurs it seems to primarily occur for learned behaviors (of acquired value) but not for innate self-initiated behaviors.

## Discussion

### The response reliability of SPNs and PV interneurons is low

In this study, we aimed to characterize the response reliability of HaR striatal neurons in freely moving animals during self-initiated and learned actions. To this end, we conducted microendoscopic Ca^2+^ imaging in freely moving mice during spontaneous movement and the different stages of learning in an operant conditioning task. The Ca^2+^ indicator GCaMP6f was expressed in a sparse neuronal population consisting predominantly of SPNs, with PV interneurons making up a smaller but substantial portion (Fig. 1-1). These subtypes represent the HaR striatal populations that receive a small number of strong afferents from the cortex (Plotkin et al., 2011) and thalamus (Johansson and Silberberg, 2020). Because the sum of fewer inputs is expected to exhibit more variability in spike timing, we predicted and found that HaR neurons exhibit low levels of response reliability.

The significant responses we observed in HaR neurons around the initiation of spontaneous movement and the presentation of task-related cues (Figs. 1, 2) are consistent with previous research on SPN and PV interneuron function. SPNs and PV-fast-spiking interneurons (FSIs) have been implicated in various aspects of movement promotion and inhibition (Gittis et al., 2011; Cui et al., 2013; Isomura et al., 2013; Jin et al., 2014; Tecuapetla et al., 2014; Perk et al., 2015; Barbera et al., 2016; Tepper and Koós, 2016; Klaus et al., 2017; Parker et al., 2018; Gritton et al., 2019). Additionally, these two neuronal populations have been linked to various aspects of learning (Perk et al., 2015; Lee et al., 2017; O’Hare et al., 2017; Martiros et al., 2018; Owen et al., 2018), with SPNs and FSIs exhibiting similar response patterns after cue presentation in conditioning tasks (Bakhurin et al., 2016; Shin et al., 2018; Peters et al., 2021). Finally, SPN responses to cue presentations have been reported to develop as training progresses (Bakhurin et al., 2016), with neurons either increasing or decreasing their activity (Bakhurin et al., 2016; Shin et al., 2018), similar to our observations here (Fig. 2 and Fig. 1-3). Thus, in both behavioral contexts, our significantly responsive cells likely belong to both striatal subtypes.

At the population level, imaged neurons showed clear responses to both self-initiated movement and the presentation of task-related cues (Figs. 1, 2). However, an examination of the signals of individual neurons revealed that a relatively small subset of neurons (approximately 30%) significantly modulated their activity following these behavioral events, while in most neurons, the average Ca^2+^ response was not significantly altered (Figs. 1*G*, 2*E*). This responsiveness rate is consistent with the findings in a recent electrophysiological study, where a similar proportion of SPNs modulated their signals near starts and ends of locomotion in freely moving mice (Fobbs et al., 2020). Previous studies quantifying the portion of SPNs coding for changes in value or for cue presentation in conditioning tasks also reported comparable percentages (between 25 and 50%) (Bakhurin et al., 2016; Shin et al., 2018). Thus, during both spontaneous movement and learning, the striatal HaR response depends on a small subset of the neurons—a functional organization that is likely to result in highly variable processing, in accordance with our observations (Figs. 3,4).

Our motivation for characterizing striatal response reliability during operant conditioning in addition to spontaneous movement was twofold. First, the dorsal striatum is known to play an essential role in motor function as well as stimulus–response learning. Analyzing the activity patterns of striatal HaR neurons within these two behavioral paradigms affords a more comprehensive view of their response reliability. Additionally, we hypothesized that learned actions, supposedly carrying a greater behavioral significance, would be accompanied by more consistent neuronal responses. However, our data did not support this hypothesis, revealing comparable levels of response reliability around self-initiated movements and task-related cues (Fig. 3).

### Sparse expression of GCaMP6 in striatal neuronal populations

Based on our previous successful implementation of a viral strategy to express GCaMP6 selectively in striatal CINs by inoculating the dorsal striatum of (Jax stock 006410) ChAT-Cre mice (Rehani et al., 2019), we cross-bred the same mouse strain with a Cre-dependent GCaMP6f mouse strain, which had recently become available (Daigle et al., 2018) with the intent to selectively target striatal CINs. However, rather than achieving a specific expression of the indicator in CINs, this strategy promoted its sparse expression in a mixture of neuronal subtypes (see qualification at the website of Jax, stock 006410). Our immunohistochemical analysis revealed that while a small proportion of GCaMP6f-expressing neurons were cholinergic, imaged cells were predominantly SPNs, with PV interneurons comprising a smaller but notable fraction (Fig. 1-1). Unfortunately, the electrophysiological properties revealed by in vivo endoscopic Ca^2+^ imaging are not sufficient to confidently distinguish the two main GCaMP-expressing subtypes in our mice—SPNs and PV interneurons. However, these two neuronal populations share electrophysiological and functional connectivity properties (Plotkin et al., 2011; Johansson and Silberberg, 2020), which likely impact the reliability of their responses and support our decision to combine them to address our questions.

CINs, representing a minority fraction of our imaged cells (Fig. 1-1), possess distinct functional connectivity properties compared to striatal HaR neurons (Johansson and Silberberg, 2020), potentially complicating the interpretation of our results. Nevertheless, given their representation of only ∼10% of the total population, their influence on the group analysis is minimal. This holds particularly true for correlation-based investigations, where the relative proportion of CIN pairs to the total number of pairs is even smaller. Notably, previous literature has demonstrated the reliability of CIN responses (Kimura et al., 1984; Raz et al., 1996; Apicella et al., 1997). Thus, the fact that the full population exhibits unreliable responses despite the inclusion of a small proportion of CINs serves to strengthen the robustness of our findings. Finally, CINs are known to respond to reward-associated stimuli with a characteristic pause in their tonic firing, often accompanied by bursting (Kimura et al., 1984; Aosaki et al., 1994; Raz et al., 1996). This phenomenon might correspond to the cessation of Ca^2+^ events underlying the negatively modulated responses observed in our study (Figs. 1, F2 and Fig. 1-3). However, it is worth mentioning that SPNs, which constitute the majority of our imaged population (Fig. 1-1), have also been reported to decrease their firing rate following the presentation of task-related cues (Bakhurin et al., 2016; Shin et al., 2018).

### Studying response reliability with sparse expression of GCaMP6 in the striatum

Our mice expressed GCaMP6f in a sparse population of striatal neurons (Fig. 1-1). This sparse expression enabled us to track and compare the responses of the same individual neurons across sessions, days, and even weeks apart. While the proportion of movement responsive neurons remained relatively stable across free movement sessions, a large fraction of neurons that significantly modulated their activity around movement initiation on the first session did not do so on the second session (Fig. 3*B*,*D*,*E*). Similarly, the rate of neurons that significantly responded to CS+ presentation gradually increased as training progressed and was stable on advanced training sessions (Fig. 2). However, the identity of significantly responsive neurons varied considerably from session to session (Fig. 3*C*,*F*). These findings, which were facilitated by the sparse expression of the indicator, suggest that the recruitment of striatal neurons to respond to various behavioral events is dynamic. In addition, the identity of neurons significantly modulating their activity around movement initiation, cue presentation or both of them is consistent with neurons being randomly recruited to respond to various behavioral events (Fig. 3*I*). Taken together, our results suggest that action representation in HaR striatal neurons, changes dynamically within and among sessions.

Importantly, the hypothesis presented in the introduction lacked a precise quantitative definition of what would be considered a reliable response, posing challenges in definitively confirming or disproving it. However, the low values of overlap in significantly responsive neurons across multiple imaging sessions (e.g., only one neuron consistently exhibited significant responsiveness around CS+ presentation in three consecutive advanced training sessions; Fig. 3*F*), along with the observed percentages of responses during a single session (e.g., neurons classified as positively modulated during movement onset responded, on average, to approximately 7% of movement initiations; Fig. 3*G*), lead us to conclude that these responses can be deemed unreliable.

### Advantages of encoding with unreliable neurons

What are the advantages of dynamically encoding a population response reliably while a majority of neurons do not participate in the encoding and while the identity of the neurons encoding constantly changes? One possibility is that the question is ill posed because we are not tracking enough parameters of the outside world. By collapsing all the neurons onto a single coarse behavior (e.g., movement initiations) we might be missing other encoded dimensions (Jensen et al., 2022; Liberti et al., 2022). While we cannot rule out that possibility, it is also possible that there are inherent advantages in dynamic encoding. The striatum is widely viewed as essential for selecting the appropriate motor plan during ongoing behavior. To accomplish this task in a constantly changing environment, it is critical for the responses of striatal neurons, and particularly those of projection neurons, to be highly flexible. A scheme in which the identity of responsive neurons changes from day to day and even between trials within the same day could simplify the encoding of new stimulus-action contingencies.

Alternatively, our data are consistent with the striatum implementing a population code, where the portion of the population activated around a behavioral event is stable, and the identities of the responsive neurons change continuously (Figs. 3, 4). Because each pallidal and/or nigral neuron receives afferents from many SPNs (Percheron et al., 1984), it may be indifferent to the identity of the projecting neurons. Similarly, while PV interneurons are few in the striatum, their dense axonal arborization extends far from the soma, with each PV interneuron synapsing onto hundreds of SPNs (Koos et al., 2004; Tepper, 2010; Berke, 2011). Thus, while it may be that the low response reliability of striatal neurons does not improve the information decoding in downstream structures, it also does not degrade it due to the highly convergent architecture of these neurons’ BG circuits (Percheron et al., 1987).

### Comparison with previous studies

Our findings of unreliable responses in striatal HaR neurons, while consistent with striatal anatomical and functional connectivity (Plotkin et al., 2011; Johansson and Silberberg, 2020), diverge from existing primate literature reporting highly reliable behavioral responses in striatal neurons, including SPNs (Apicella et al., 1992; Cromwell and Schultz, 2003). Notably, these primate studies relied on highly controlled behaviors, usually consisting of over-trained, head-fixed animals performing a constrained set of movements. In contrast, our recordings were conducted in freely moving mice engaged in self-initiated and naturalistic behaviors. While HaR unreliability was maintained when we focused on more stereotypical, controlled movements (e.g., ipsi- and contralateral turns, grooming; Fig. 4) and learning states (Fig. 3 and Fig. 2-1), it is possible, as we discussed above, that response variability arises, at least partially, from the uncontrolled nature of the behavior rather than an intrinsic unreliability of the neuronal responses. Alternatively, the discrepancy could stem from the fundamentally different behavioral conditions between the primate studies and our work, reflecting an inherent difference in the organization of striatal HaR responses during highly trained task-related behaviors compared to spontaneous, self-initiated movements in a freely moving animal. While this is an essential caveat of our experimental design, it also allowed us to study neuronal activity in a more naturalistic setting.

The body of research investigating the reliability of striatal responses in mice is limited. Nevertheless, existing studies using electrophysiological recordings (Kubota et al., 2009) and Ca^2+^ imaging (Sheng et al., 2019) support the notion that task proficiency shapes SPN responses. As training progresses, the pattern of SPN neural activity undergoes qualitative changes, with activity peaks shifting to different parts of the task (Kubota et al., 2009; Sheng et al., 2019). Moreover, in the early stages of training, SPN neural activity around task-related motor behaviors displays considerably more trial-to-trial variability compared to extensively trained mice (Kubota et al., 2009; Sheng et al., 2019).

Another significant factor possibly influencing the reliability of observed SPN responses is the duration of recording and the number of repetitions conducted within each session. A recent study in which rats performed distinct, highly stereotyped forelimb movements found that the mean activity profiles of individual striatal neurons relative to different stages of the movement were highly consistent across multiple recording days (Jensen et al., 2022). Importantly, this study utilized an automated data collection system within the home cage, allowing for the collection of extensive data comprising numerous trials and hours of recording per day. It is plausible that SPNs display trial-to-trial response variability that gets averaged out when the activity from each recording day is combined over a large amount of time and repetitions. In contrast, our data collection approach involved sampling neural activity for a shorter duration of 30–45 min each day. This method is more sensitive to trial-to-trial variability, evaluating response reliability on a different time scale.

### Ca^2+^ imaging versus electrophysiology

Another caveat inherent to our approach is that Ca^2+^ imaging signals are generally sparser and less reliable than electrophysiological recordings (Wei et al., 2020). Ca^2+^ imaging relies on the detection of changes in intracellular Ca^2+^ levels—a slow and indirect indicator of neural activity. Consequently, the ability to detect single action potentials in a Ca^2+^ imaging signal is highly dependent on the neuron’s spiking pattern and its recent activity. Factors such as the Ca^2+^ buffering properties and the expression levels of genetically encoded Ca^2+^ indicators, which may not be uniform across the imaged population, could also affect the ability to detect every spike. Although these limitations can restrict our access to the true neural responses hindering our ability to assess the reliability of the neurons, Ca^2+^ imaging remains a valuable technique for studying the activity of genetically defined neural populations. The spatial information provided by our microendoscopic Ca^2+^ imaging data, which cannot be accessed using electrophysiological methods, enabled us to trace multiple individual neurons acting simultaneously and evaluate the reliability of their responses across multiple imaging sessions and behavioral contexts.

### Correlations among HaR neurons

Next, theorizing that the unreliable responses might be compensated for by co-activation, we exploited our ability to simultaneously image dozens of striatal neurons and explored their signal and noise correlations in different behavioral states. We found that pairwise signal correlations in HaR striatal neurons are independent of the distance between them, with similar results during movement and rest, as well as near cue presentation at different stages of conditioning (Fig. 5). These findings are in line with previous in vitro studies, which demonstrated that nearby SPNs (making up the majority of our neurons, Fig. 1-1) receive very different inputs (Kincaid et al., 1998; Zheng and Wilson, 2002) and therefore should not be more correlated than distant ones.

While consistent with striatal connectivity, the distance independence of pairwise correlations (Fig. 5) in our population contrasts with prior reports of spatially compact SPN clusters in the striatum (Barbera et al., 2016; Parker et al., 2018; Shin et al., 2020). Importantly, these results were derived through the simultaneous microendoscopic imaging of thousands of SPNs, likely including a substantial background signal arising from out-of-focus somata and neuropil. Neuropil signals in other striatal subtypes have been found to exhibit high spatial correlations and show different kinetics from somatic signals (Rehani et al., 2019; Legaria et al., 2021)—a result that we reproduced here for HaR striatal neurons (Fig. 5-1). Additionally, background neuropil correlations in our data show a strong distance dependence, decaying steeply as the distance grows from 0 to 200 µm (Fig. 5-1*B*). Two recent papers using soma-targeted Ca^2+^ indicators to investigate the sources of artifactual correlation across neurons in microendoscopic imaging demonstrated that soma tagging is ineffectual in removing spurious correlations (Chen et al., 2020; Shemesh et al., 2020). This suggests that the uncontrolled contamination of the somatic signals that leads to spurious correlations arises largely from other somata that are above or below a given soma but within its depth of imaging field. A corollary of these findings is that the more somata there are in a volume being imaged, the more artifactual correlations there will be. It is thus possible that the discrepancy between our observations and previous results emerges from spatially correlated neuropil activation and out-of-focus somata contaminating somatic signals in a dense SPN population and introducing spurious, distance dependent correlations.

Further supporting our proposed explanation, pairwise signal correlations in striatal HaR neurons were, on average, weaker by orders of magnitude than previously reported values (Fig. 5). Because these studies used both slow and fast GCaMP6 variants (Barbera et al., 2016; Shin et al., 2020), it is unlikely that the discrepancy stems from differences in the kinetics of the indicator. However, if previously reported somatic correlations include spurious correlations originating from the out-of-focus somata and neuropil signal as we suggest, both of which are expected to be weaker in a sparse population, then we expect the correlation values in our data to be lower. We propose that the sparse expression of the Ca^2+^ indicator in our mice (Fig. 1-1) produces a weak neuropil signal and less out-of-focus somatic signals compared to those arising from densely expressing populations. Consequently, the background signal introduces less spurious correlations between somata, decreasing pairwise correlation values and eliminating their distance dependence. While we cannot fully address this point based on our data, which do not target SPNs exclusively, we believe that the concerns raised by our results merit consideration.

Finally, we investigated trial-to-trial correlations between pairs of striatal HaR neurons by calculating their JPSTHs around spontaneous movement onset and cue presentation. For self-initiated movements, we found that any covariation beyond common modulations in the average rate of Ca^2+^ transients is most likely explained by variability in the movement itself, rather than the temporal coupling of responsive neurons (Fig. 6). However, with respect to learned associations, the JPSTH analysis suggests that there may be additional components of a dynamic organization into temporally coupled subsets of neurons.

## Conclusions

In conclusion, our analysis of sparsely labeled SPNs and PV interneurons, which represent striatal HaR neurons, in freely moving mice demonstrated that individual neurons are recruited in a stochastic and dynamic fashion to respond to specific actions, presenting low response reliability. The JPSTH analysis suggests inherent differences in the collective dynamics during learning and innate behaviors, possibly signifying that the coactivation during task learning is more “meaningful” to the neural code than around spontaneous movement. We speculate that the low degree of response reliability affords SPNs more flexibility in responding to a battery of potential actions and/or various multiplexed encodings. On the other hand, because of the large degree of convergence in the pallidum and substantia nigra pars reticulata (Percheron et al., 1987, 1994; Bolam et al., 1993), this lack of reliability will likely not affect the constancy of downstream pallidal or nigral representations.

## Synthesis

Reviewing Editor: Arvind Kumar, KTH Royal Institute of Technology Decisions are customarily a result of the Reviewing Editor and the peer reviewers coming together and discussing their recommendations until a consensus is reached. When revisions are invited, a fact-based synthesis statement explaining their decision and outlining what is needed to prepare a revision will be listed below. The following reviewer(s) agreed to reveal their identity: NONE.

The manuscript provides new interesting data about information representation in the population activity of striatal neurons in behaving mice. Overall the reviewers think that the manuscript is already in a very good shape. They have made a few suggestions that should be incorporated in the revised version.

Specifically reviewers would like to see an explanation of the probabilistic approach you used to track neurons over time and the rationale for the conditioning paradigm. In addition, one of the reviewers also raised a point about the specific mouse line used and that issue should be addressed.

The comments from the two reviewers are appended below. We look forward to reading the revised version.

Reviewer #1

The paper presents interesting data on neuronal activity in the striatum of freely moving mice using calcium imaging with sparse labeled cells employing GcamP6f. The authors utilize a classic conditioning paradigm to assess neural responses in the striatum, revealing that changes in population activity provide a more reliable representation of motor behavior than individual neurons. This trend is consistent across various spontaneous and learned motor behaviors. The data is well-analyzed and effectively conveys the message.

Major:

In lines 65-68, the text could be clarified as it initially suggests a comparison of different neuron types but later focuses only on hyperpolarized at rest neurons.

The authors should provide a more detailed explanation of the probabilistic approach for cell registration to track neurons over time.

Including an illustrative example of how neurons were tracked longitudinally would enhance clarity.

Mentioning the rationale for choosing a conditioning paradigm to study motor behavior in the discussion would be beneficial.

Minor:

Correct the spelling of "Carprofen" in line 110.

Correct the spelling of "Xylazine" in line 111.

Ensure that axis titles in Figure 1, Panels E-F, are visible.

Verify the units for acceleration for accuracy.

Reviewer #2

I have seen this paper before when it was in review in a different journal, and I am pleased to see this paper has been significantly revised and addressed my prior concerns. As such, I won't reiterate critiques from the prior version I saw. I think the paper is ready to publish.

I will say that re-reading this paper gave me new thoughts about the main elephant in the room/paper: the expression pattern they obtained with Chat-Cre line. I understand the challenge they experienced with their Chat-Cre line and the unexpected expression pattern they obtained. I also appreciate how they clearly describe this issue in their Discussion. However, I remain confused about whether they had a specific issue with the stock they received or the breeding in their lab, or whether they identified a more general issue with Jax line 006410. This seems important I searched for this line and it appears to have been used (at least) 92 times in the literature for targeting ChAT + neurons (https://scicrunch.org/resources/data/record/nlx_154697-1/IMSR_JAX:006410/resolver/mentions?q=006410&i=rrid::imsr_jax:006410). Can the authors clarify if they think this is a specific issue they experienced or a more general issue with this line?

## Author Response

Synthesis of Reviews: Computational Neuroscience Model Code Accessibility Comments for Author (Required): N/A Synthesis Statement for Author (Required): Synthesis The manuscript provides new interesting data about information representation in the population activity of striatal neurons in behaving mice. Overall the reviewers think that the manuscript is already in a very good shape. They have made a few suggestions that should be incorporated in the revised version. Specifically reviewers would like to see an explanation of the probabilistic approach you used to track neurons over time and the rationale for the conditioning paradigm. In addition, one of the reviewers also raised a point about the specific mouse line used and that issue should be addressed. The comments from the two reviewers are appended below. We look forward to reading the revised version. We are happy that you and the reviewers are enthusiastic about our manuscript. As explained below, we have addressed all of the reviewers suggestions, particularly with respect to the method to track neurons across sessions, which has resulted in a new panel in Figure 3 (panel A) and an expanded explanation of the method in the text. We have also clarified in more detail how the mouse strains we used led to spurious expression of GCaMP6f. Reviewer #1 The paper presents interesting data on neuronal activity in the striatum of freely moving mice using calcium imaging with sparse labeled cells employing GcamP6f. The authors utilize a classic conditioning paradigm to assess neural responses in the striatum, revealing that changes in population activity provide a more reliable representation of motor behavior than individual neurons. This trend is consistent across various spontaneous and learned motor behaviors. The data is well-analyzed and effectively conveys the message. Major: 2 In lines 65-68, the text could be clarified as it initially suggests a comparison of different neuron types but later focuses only on hyperpolarized at rest neurons. We are grateful for the reviewer's overall positive appraisal of the manuscript. The purpose of the cited paragraph was to introduce the question and to justify our focus on HaR neurons since, as we explain in the following lines, spontaneously active striatal neurons are known to be highly reliable. However, we agree that it can give the impression that we had compared the response reliability of spontaneously active striatal neurons and HaR neurons. To clarify this point, we now explicitly state in line 68 that our study focuses on HaR neurons. Lines 69-73 now read: "Therefore, the striatum provides a good testing ground for the relationship between input integration by individual neurons and their response reliability: autonomous pacemakers (CINs and LTSIs) should be highly reliable in their representation of stimuli and actions, while HaR neurons (SPNs and PV interneurons, the focus of the current study) should be less reliable." The authors should provide a more detailed explanation of the probabilistic approach for cell registration to track neurons over time. Including an illustrative example of how neurons were tracked longitudinally would enhance clarity. To track neurons across imaging sessions, we used CellReg - a probabilistic approach for cell registration across multiple sessions in Ca2+ imaging data. CellReg is a well-established algorithm widely adopted within the Ca2+ imaging community, and we used the implementation provided by the algorithm's developers (Sheintuch et al., Cell Reports, 2017). In the revised version of the manuscript, we clarified this point and included a short description of the algorithm as well as the criteria we used in applying it (line 139): "To track individual cells across imaging sessions, we used CellReg - a well-established probabilistic approach to automatically register cells across multiple imaging sessions in Ca2+ imaging data (Sheintuch et al., 2017). Briefly, we used CNMF-E to generate spatial footprints for all detected cells in the relevant imaging sessions. Footprints from the earliest session served as a reference map. Footprints from subsequent sessions were aligned to this reference by correcting for rotational and translational differences. The algorithm yields a list of cells, each representing the same cell over multiple sessions. For each set of cells representing the same cell in different imaging sessions, the probability that they all belong to the same cell was 3 calculated (Psame). We included a cell in our analysis if Psame > 0.5 and the distance between the centroids across sessions was < 14 µm. To quantify the registration confidence for each cell, CellReg provides a strict register score index (ranging between 0 and 1), which factors in the registration certainty of all cell pairs. In all cases, the average register score was > 0.97." Finally, we have included in the revised manuscript, a new figure panel that visually demonstrates the algorithm's performance on our data (Fig. 3A). Mentioning the rationale for choosing a conditioning paradigm to study motor behavior in the discussion would be beneficial. We had two reasons for studying the reliability of striatal HaR neurons in a conditioning paradigm in addition to during spontaneous movement. First, because motor function and stimulus-response learning are two of the main functions of the striatum, we reasoned that characterizing striatal reliability during both types of behavior would provide a more complete picture. Additionally, we hypothesized that there may be a difference in the reliability of striatal responses for spontaneous and learned actions. Specifically, we postulated that learned actions, presumably holding a higher behavioral value, would be accompanied by more reliable responses (which ended up not aligning with our findings). We briefly touched on the latter point in the introduction (lines 83-86). However, we agree that this should be elaborated upon and do so in the discussion of the revised manuscript. Lines 723-731 now read: "Our motivation for characterizing striatal response reliability during operant conditioning in addition to spontaneous movement was two-fold. First, the dorsal striatum is known to play an essential role in motor function as well as stimulus-response learning. Analyzing the activity patterns of striatal HaR neurons within these two behavioral paradigms affords a more comprehensive view of their response reliability. Additionally, we hypothesized that learned actions, supposedly carrying a greater behavioral significance, would be accompanied by more consistent neuronal responses. However, our data did not support this hypothesis, revealing comparable levels of response reliability around self-initiated movements and task related cues (Fig 3)." Minor: Correct the spelling of "Carprofen" in line 110. Corrected. 4 Correct the spelling of "Xylazine" in line 111. Corrected. Ensure that axis titles in Figure 1, Panels E-F, are visible. Corrected. Verify the units for acceleration for accuracy. [g] is the gravitational constant 9.81 m/s2 . This is now explicitly stated in the legends of figures 1, 1-2, 2, 2-1, 2-2 and 4. Reviewer #2 I have seen this paper before when it was in review in a different journal, and I am pleased to see this paper has been significantly revised and addressed my prior concerns. As such, I won't reiterate critiques from the prior version I saw. I think the paper is ready to publish. We thank the reviewer for this assessment and are pleased that we were able to answer all of their previous concerns. This and other reviewers' important comments have greatly improved the manuscript. I will say that re-reading this paper gave me new thoughts about the main elephant in the room/paper: the expression pattern they obtained with Chat-Cre line. I understand the challenge they experienced with their Chat-Cre line and the unexpected expression pattern they obtained. I also appreciate how they clearly describe this issue in their Discussion. However, I remain confused about whether they had a specific issue with the stock they received or the breeding in their lab, or whether they identified a more general issue with Jax line 006410. This seems important I searched for this line and it appears to have been used (at least) 92 times in the literature for targeting ChAT + neurons (https://scicrunch.org/resources/data/record/nlx_1546971/IMSR_JAX:006410/resolver/ mentions?q=006410&i=rrid::imsr_jax:006410). Can the authors clarify if they think this is a specific issue they experienced or a more general issue with this line? We believe that what we have encountered is a general issue with this ChAT-Cre strain. The non-selective GCaMP6f expression in our cross-bred mice is likely due to the presence of the 5 frt-flanked neo cassette in the Jax line 006410, which can result in the ectopic expression of Cre. This qualification has since appeared on the Jackson website (but was not at the time when we began our experiments with these mice). Since identifying this issue, we have reached out to colleagues around the world (e.g., Karolinska, NYU) who report having experienced similar problems with their ChAT-Cre mice as well as Cre strains in general. In our hands, the issues arise when this ChAT-Cre mouse line is cross-bred with reporter mouse strains, but not when viral strategies are used in adult mice: when we inoculated the dorsal striatum of these mice with AAVs harboring a Cre-dependent GCaMP6s construct we got selective expression in striatal cholinergic interneurons (see Fig. 1A in our paper, Rehani et al. eNeuro 2019) . Thus, it could be that in many - but certainly not all - of the 92 studies you mention the viral strategy was used and so no ectopic expression was encountered. We have clarified this issue more explicitly in the Discussion, as follows (lines 734-740): "Based on our previous successful implementation of a viral strategy to express GCaMP6 selectively in striatal CINs by inoculating the dorsal striatum of (Jax stock 006410) ChAT-Cre mice (Rehani et al., 2019), we cross-bred the same mouse strain with a Cre-dependent GCaMP6f mouse strain, which had recently become available (Daigle at al., 2018) with the intent to selectively target striatal CINs. However, rather than achieving a specific expression of the indicator in CINs, this strategy promoted its sparse expression in a mixture of neuronal subtypes (see qualification at the website of Jax, stock 006410).
