# Supplementary figures and images for: Striatal Neurons Are Recruited Dynamically into Collective Representations of Self-Initiated and Learned Actions in Freely Moving Mice
Source: eNeuro. 2024 Jan 10;11(1):ENEURO.0315-23.2023. doi: 10.1523/ENEURO.0315-23.2023 (PMC11057506; doi:10.1523/ENEURO.0315-23.2023)

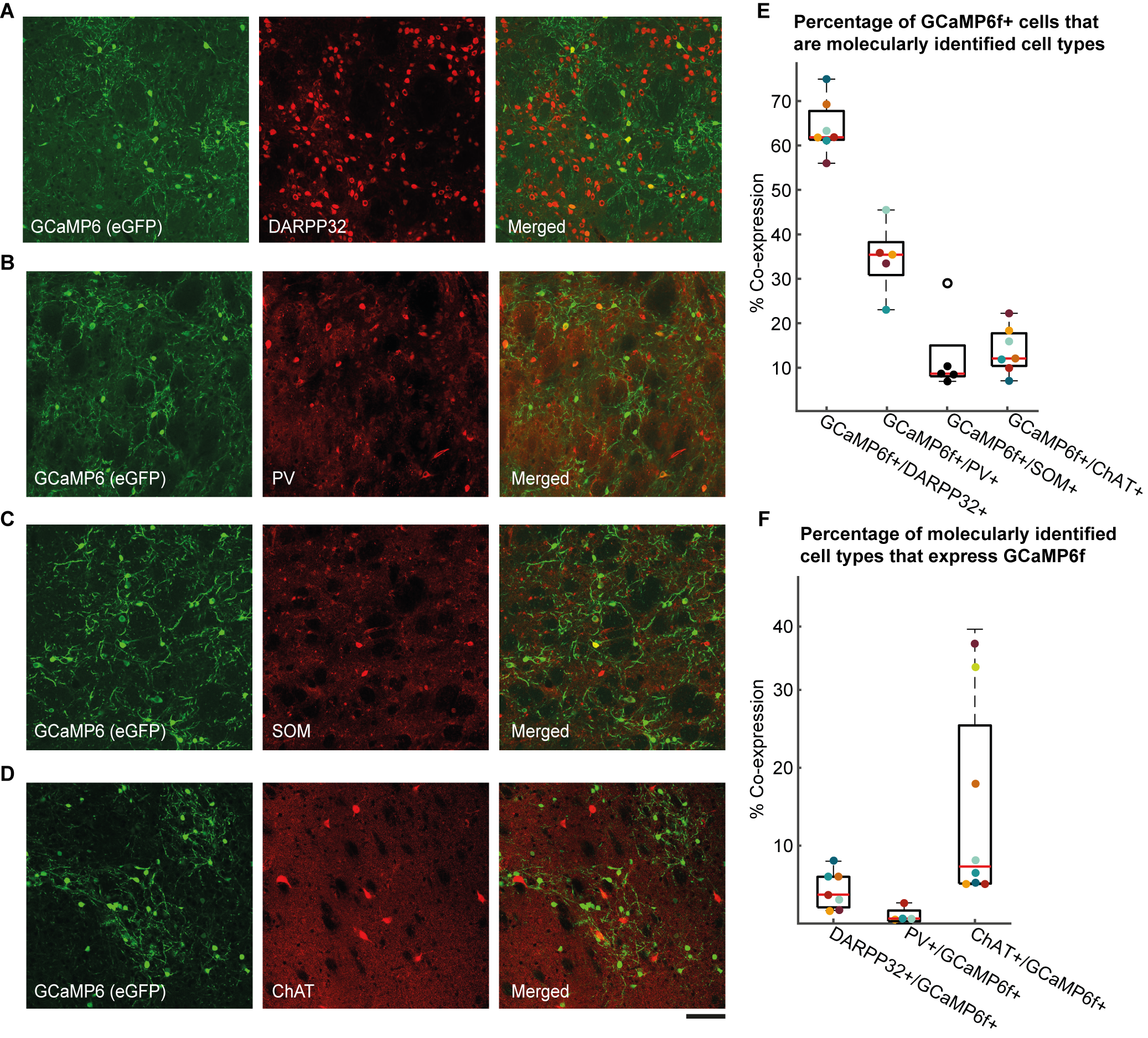

Supplement: Figure 1-1 — Striatal neurons in the sparse GCaMP mouse are a mixture of neuronal types with an SPN majority. A: Immunohistochemical analysis of dorsal striatum of sparse GCaMP mice demonstrates that a large portion of GCaMP6f expressing cells express DARPP32. B: Same as A for the co-expression of GCaMP6f and parvalbumin (PV). C: Same as A for the co-expression of GCaMP6f and somatostatin (SOM). D: Same as A for the co-expression of GCaMP6f and ChAT. Scale bar = 100 μm (A-D). E: Rates of co-expression of GCaMP6f and markers for various neuronal subtypes in the striatum. 64±2.3% (mean ± S.E.M; range 56-74.9%, N=7 mice) of GCaMP6f-expressing neurons co-express DARPP32, an established SPN marker (Ouimet et al., 1998); 34.64±3.58% (mean ± S.E.M; range 23-45.5%, N=5 mice) co-express PV; and 8.6±0.7% [mean ± S.E.M; range 6.9-10.3%, N=4 mice; one outlier (29%) was excluded from the analysis] co-express SOM;13.9±2% (mean ± S.E.M; range 7-22.2%, N=7 mice) of GCaMP6f-expressing cells exhibited immunoreactivity to ChAT; Importantly, the analysis for the co-expression of SOM and GCaMP6f was conducted on sparse GCaMP mice that were not included in the microendoscopic experiments. F: Rates of co-expression of markers for various neuronal subtypes in the striatum and GCaMP6f exhibit that SPN and PV neurons are represented sparsely among the GCaMP6f-expressing neurons. 4.33±0.91% (mean ± S.E.M; range 1.68-8%, N=7 mice) of DARPP32-expressing neurons co-express GCaMP6f; 0.99±0.58% (mean ± S.E.M; range 0-2.67%, N=4 mice) of PV-expressing neurons co-express GCaMP6f; and 14.82±4.75% [mean ± S.E.M; range 5-37.78%, N=8 mice] of ChAT-expressing neurons co-express GCaMP6f; Each data point represents the co-expression rate for a single mouse. Data points with the same color are from the same individual mouse. Black points represent data from sparse GCaMP mice that were not included in the microendoscopic experiments. Outliers are marked by empty circles. Red line is the median. Box edges are 25th and 75t [file eneuro-11-ENEURO.0315-23.2023-s002.tif]

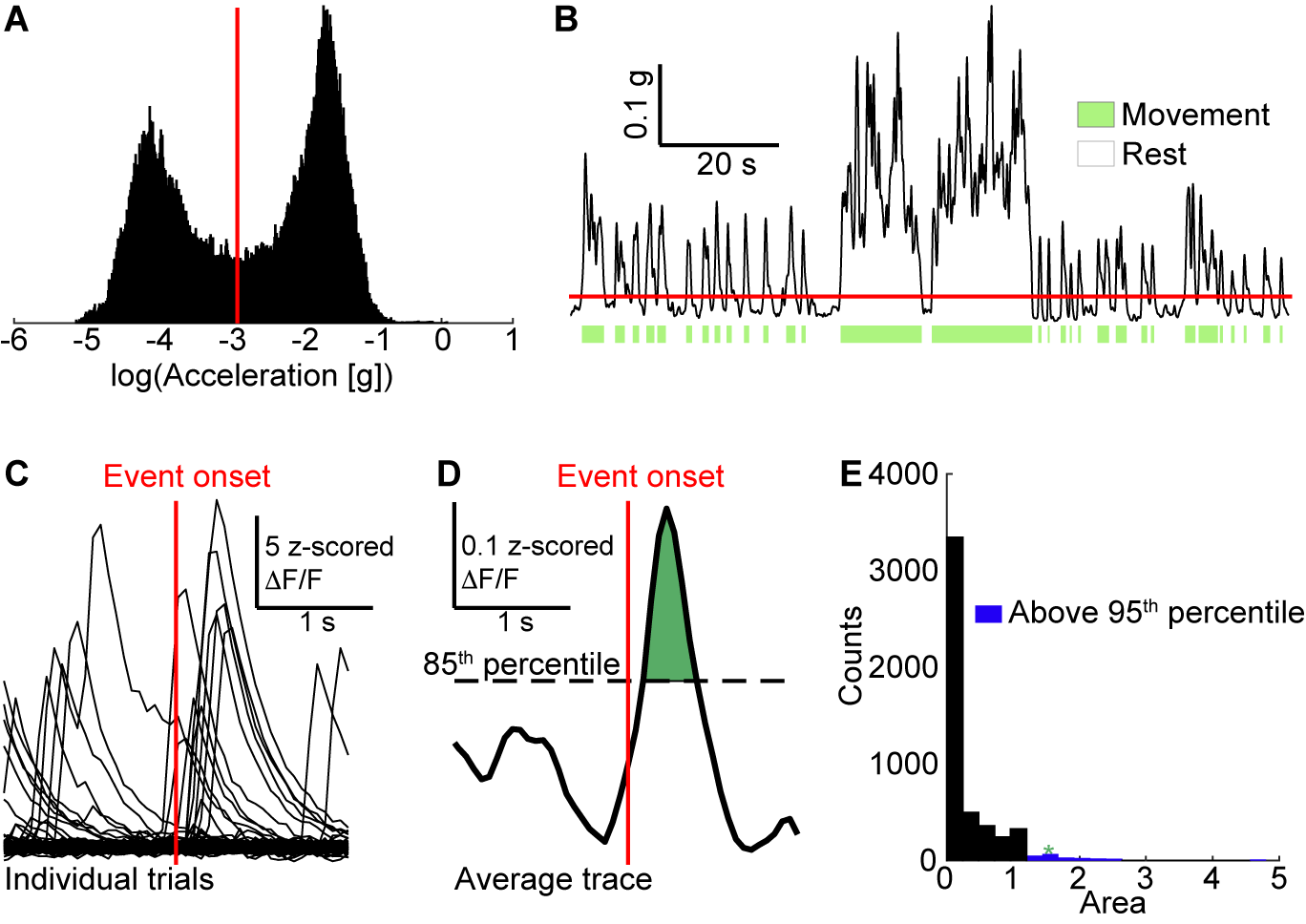

Supplement: Figure 1-2 — Detection of movement onset and offset times and determination of response significance A: Distribution of total body acceleration values from a single session in a representative mouse. Red line represents the threshold, manually set as the middle point between the two peaks in the bimodal distribution. g in the scale bar is the gravitational constant 9.81 m/s2. B: Total body acceleration trace for the same session. Red line represents the threshold. Green bars mark movement times. C: Ca2+ activity of a representative neuron around a behavioral event for various trials. Red line marks event onset. D: Average response of the same neuron. Dashed line represents an 85th percentile threshold. The statistic for significance testing is the area between the average trace and the threshold (green). E: Null distribution of area values generated by bootstrapping. The area for which null-hypothesis is rejected is in blue. The empirical area value for the given neuron is marked by the green asterisk. Download Figure 1-2, TIF file. [file eneuro-11-ENEURO.0315-23.2023-s003.tif]

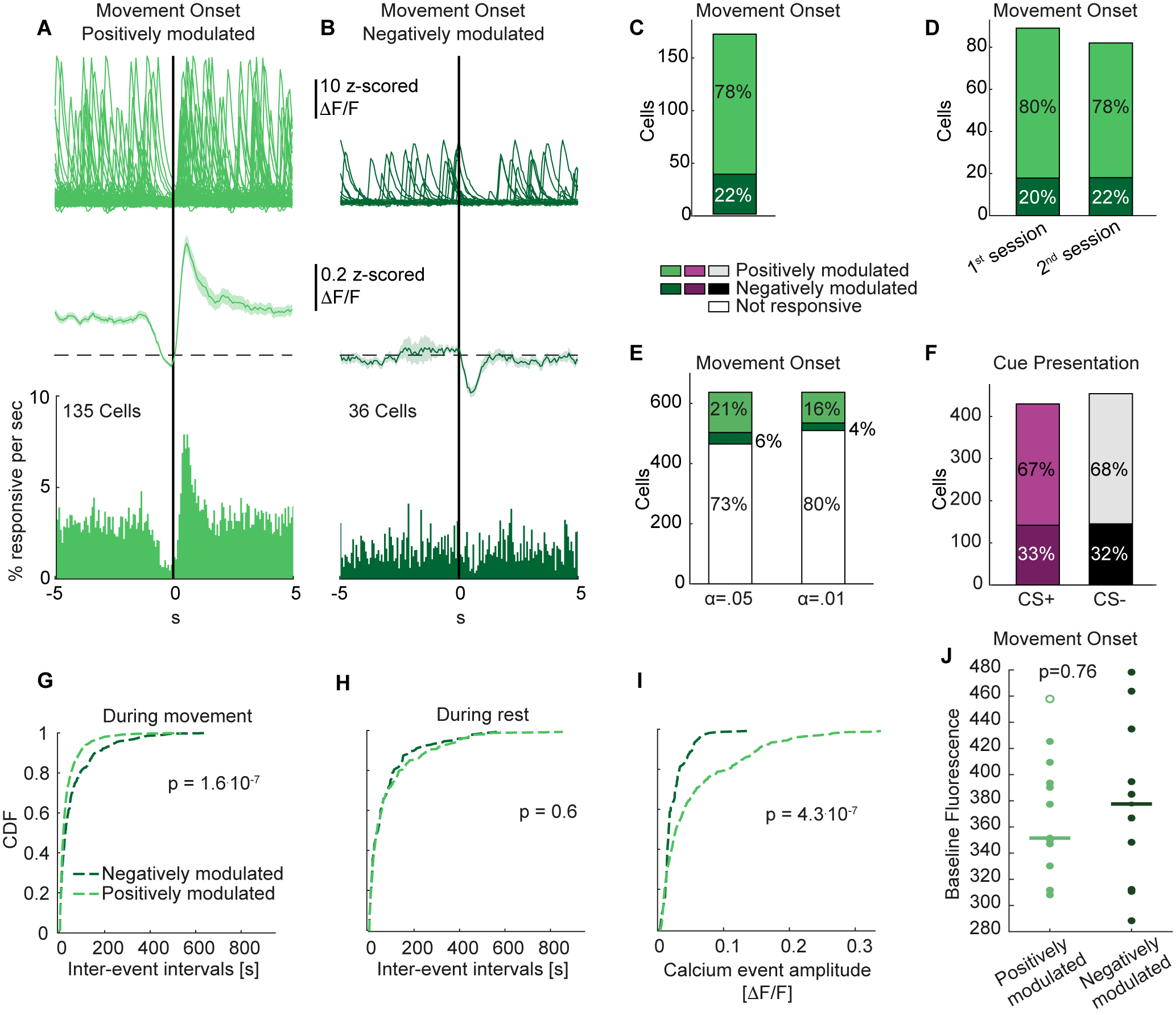

Supplement: Figure 1-3 — Two types of responses around self-initiated movement. A: Ca2+ activity around movement onset of a representative positively modulated neuron (top). Each trace represents the activity around a single movement initiation event. The average Ca2+ activity across the population of positively modulated neurons (middle). Shaded areas represent S.E.M. PSTH of Ca2+ events around movement onset in the population of positively modulated neurons (bottom). B: Same as A, for neurons that are negatively modulated around movement onset. C: The percentage of positively (light) and negatively (dark) modulated neurons, out of the population of neurons significantly modulated around movement onset. D: Rates of neurons positively and negatively modulating their Ca2+ signals following movement onset on the first (left) and second (right) free movement imaging sessions. E: Rates of imaged neurons positively and negatively modulating their Ca2+ signals following movement onset for different significance levels. F: Same as C, for neurons significantly modulated around the presentation of task-related cues. G: The CDFs of inter-event intervals for positively (light green) and negatively (dark green) modulated neurons during movement. RST. H: Same as F, during rest. RST. I: CDFs of Ca2+ event amplitudes in positively (light green) and negatively (dark green) modulated neurons during rest. RST. J: Baseline fluorescence values for positively and negatively modulated neurons. Each data point is the average value across all neurons of the relevant category in the given mouse and session. Empty circles represent outliers. RST. Download Figure 1-3, TIF file. [file eneuro-11-ENEURO.0315-23.2023-s004.tif]

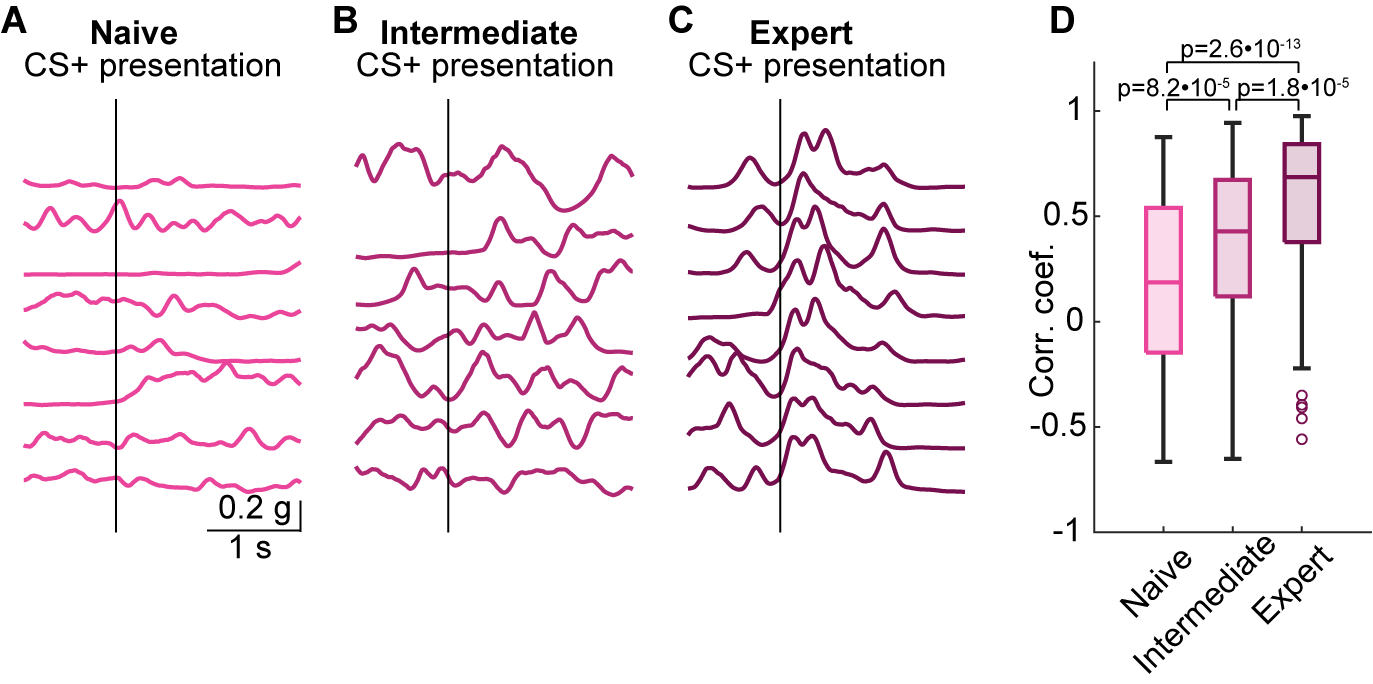

Supplement: Figure 2-1 — Mice acceleration profiles following CS+ presentation become more stereotyped as training progresses. A: Example consecutive total body acceleration traces from a representative mouse around CS+ presentation on naïve training sessions. Each trace represents a single trial. B: Same as A, for intermediate training sessions in the same mouse. C: Same as A, for expert training sessions in the same mouse. D: Box plot of the correlations between the total body acceleration around CS+ presentation in each trial and the average correlation trace across all trials in the same session for naïve, intermediate and expert training sessions. Data from 6 mice are pooled together for each training stage. Outliers are marked by empty circles. The middle line in each box is the median. Box edges are 25th and 75th percentile. Whiskers extend to the most extreme data points not considered outliers. RST. g in the scale bar is the gravitational constant 9.81 m/s2. Download Figure 2-1, TIF file. [file eneuro-11-ENEURO.0315-23.2023-s005.tif]

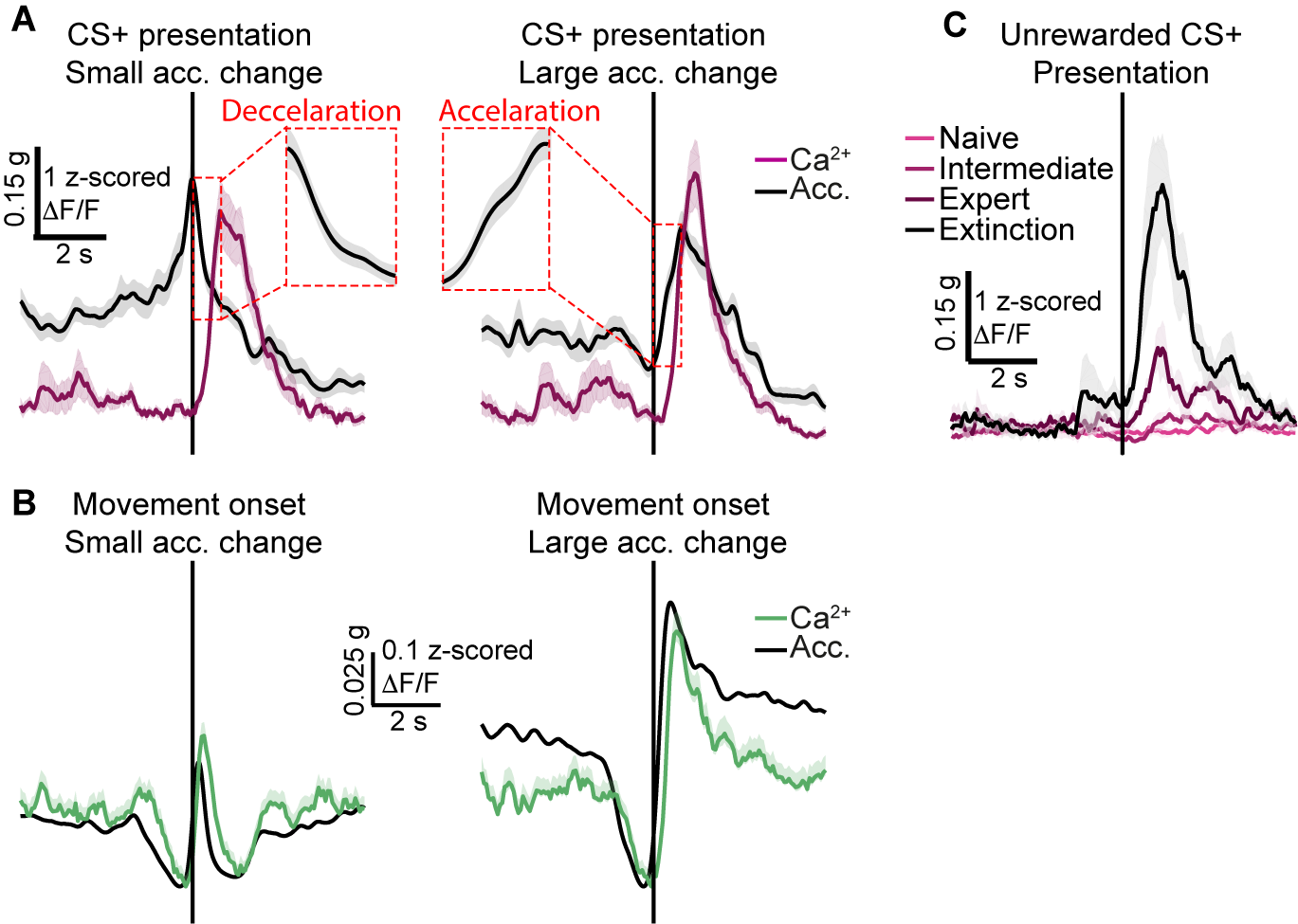

Supplement: Figure 2-2 — Responses to cue presentation are not determined by total body acceleration or reward delivery. A: Average Ca2+ activity across the neuronal population (pink) and total body acceleration (black) around CS+ presentations with small (left, 1st quartile) and large (right, 4th quartile) acceleration changes. B: Same as A, around movement initiations. Ca2+ activity shown in green. Total body acceleration shown in black. C: Average Ca2+ activity across the population for unrewarded CS+ presentations on various stages of conditioning (light to dark pink), and for the first 10 CS+ presentations on the first extinction session (black). Shaded areas represent S.E.M. g in the scale bar is the gravitational constant 9.81 m/s2. Download Figure 2-2, TIF file. [file eneuro-11-ENEURO.0315-23.2023-s006.tif]

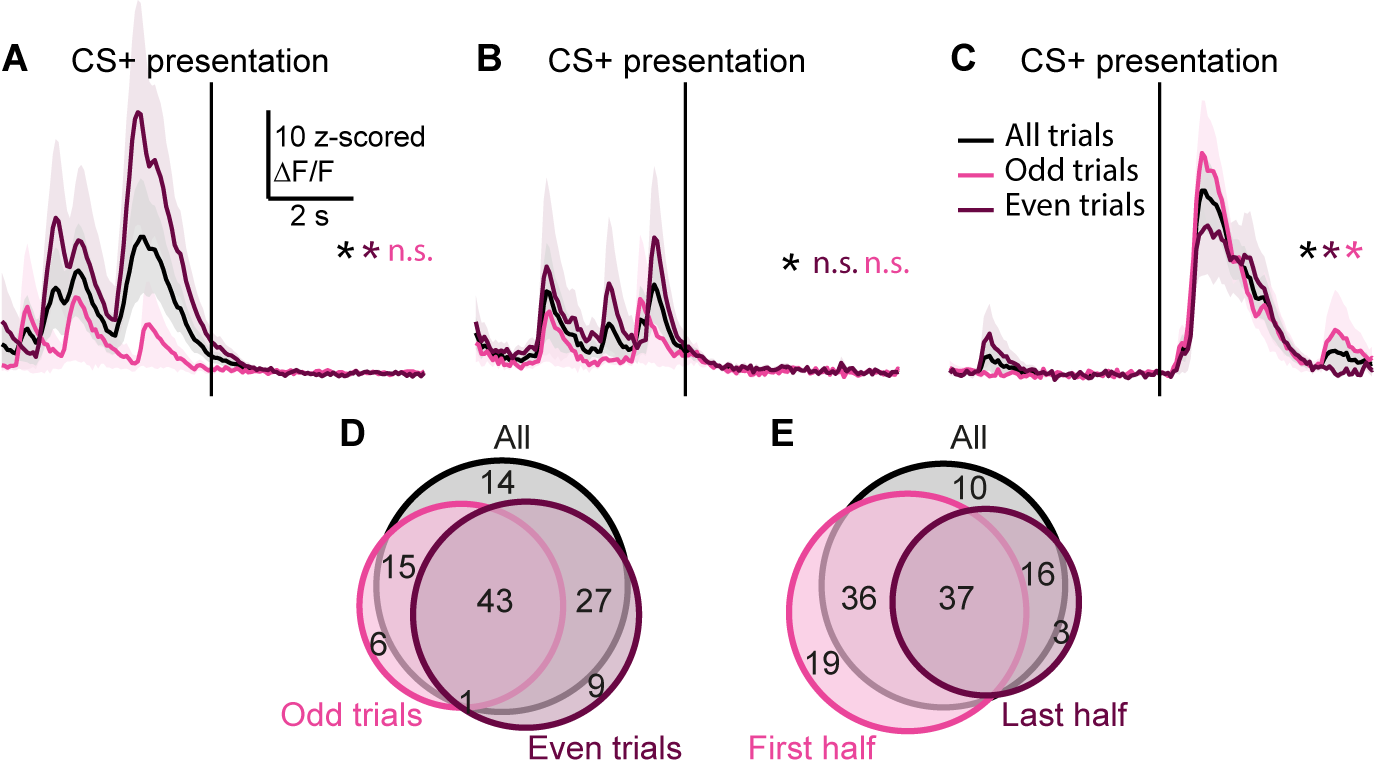

Supplement: Figure 3-1 — HaR neurons are dynamically recruited within a single session and around a consistent behavior. A: Average Ca2+ activity for an example neuron around CS+ presentation across all trials (black), odd trials only (light pink) and even trials only (dark pink). Shaded areas represent S.E.M. Asterisks mark significant responses with black, light pink and dark pink corresponding to all, odd and even trials, respectively. B-C. Same as A, for different neurons. D. Venn diagram of significantly responsive neurons when bootstrapping-based significance testing was performed using all trials (black), odd trials only (light pink) or even trials only (dark pink). E. Same as D, for all trials (black), the first (light pink) and the last half of trials (dark pink). Download Figure 3-1, TIF file. [file eneuro-11-ENEURO.0315-23.2023-s007.tif]

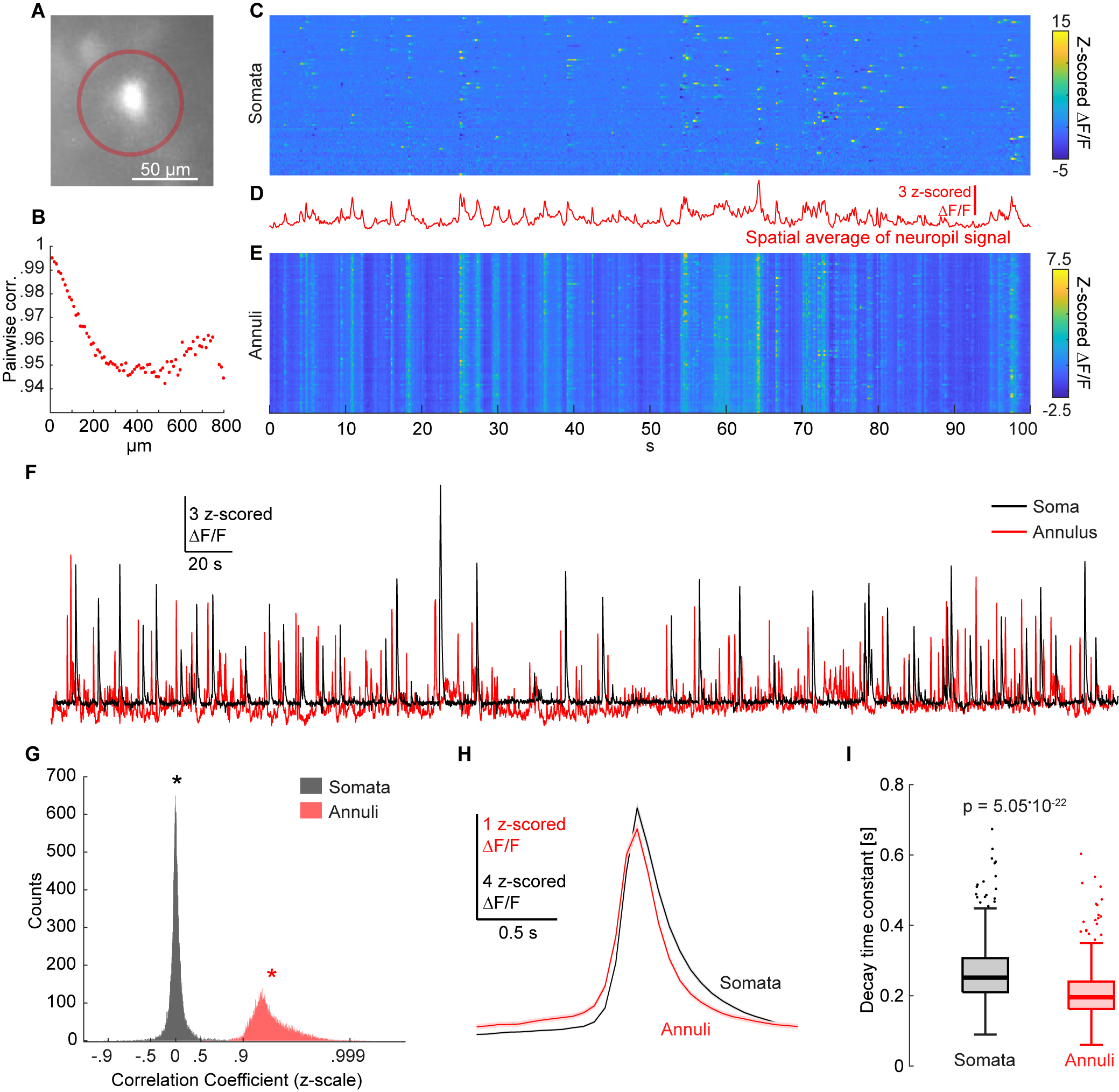

Supplement: Figure 5-1 — Neuropil signal is highly correlated in space and displays different kinetics then the somatic signals. A. Illustration of the sampling of a soma and the surrounding annular region of interest (red). B. Pairwise correlations between annular signals as a function of the distance between neuronal centers for all pairs of co-imaged neurons. Each point is the average correlation across all neuronal pairs belonging to the relevant 10 μm distance bin. C. Color-coded matrix of the fluctuations in fluorescence as a function of time in 80 somata detected in a single free movement session in a single mouse, with each row representing an individual soma. D. Spatial average of signals from all annuli presented in D. E. Same as B, with each row representing the signal of the corresponding annulus. F. Ca2+ signals from a soma-annulus pair. G. Distribution of pairwise correlation coefficients for 21,427 simultaneously imaged soma-soma and annulus-annulus pairs. Stars mark the means (0.023 and 0.96 for somata and annuli, respectively). I. Average Ca2+ signal from the soma and its corresponding annulus averaged over 429 soma-annulus pairs triggered on the somatic Ca2+ events. Shaded areas mark S.E.M. H. Boxplot of decay time constants for somatic and annular Ca2+ signals. The bold line is the median and the whiskers are the 25th and 75th percentiles. Dots represent outliers. Download Figure 5-1, TIF file. [file eneuro-11-ENEURO.0315-23.2023-s009.tif]
