# Supplementary material for: Striatal Neurons Are Recruited Dynamically into Collective Representations of Self-Initiated and Learned Actions in Freely Moving Mice
Source: eNeuro. 2024 Jan 10;11(1):ENEURO.0315-23.2023. doi: 10.1523/ENEURO.0315-23.2023 (PMC11057506; doi:10.1523/ENEURO.0315-23.2023)
Supplement: Table 3-2 — Time between the two free movement sessions for the various mice. Download Table 3-2, DOC file. [file eneuro-11-ENEURO.0315-23.2023-s008.doc]

| Mouse # | 1 | 2 | 3 | 4 | 5 | 6 | 7 |
| --- | --- | --- | --- | --- | --- | --- | --- |
| Days between sessions | 15 | 6 | 29 | 3 | 3 | 1 | 1 |
| **Table 3-2. Time between the two free movement sessions for the various mice.** | | | | | | | |
